# Shifts Between and Among Populations of Wheat Rhizosphere *Pseudomonas*, *Streptomyces* and *Phyllobacterium* Suggest Consistent Phosphate Mobilization at Different Wheat Growth Stages Under Abiotic Stress

**DOI:** 10.3389/fmicb.2019.03109

**Published:** 2020-01-22

**Authors:** Claudia Breitkreuz, François Buscot, Mika Tarkka, Thomas Reitz

**Affiliations:** ^1^Department of Soil Ecology, UFZ – Helmholtz Centre for Environmental Research, Halle/Saale, Germany; ^2^German Centre for Integrative Biodiversity Research (iDiv) Halle-Jena-Leipzig, Leipzig, Germany

**Keywords:** climate change, agriculture, wheat, PGPR, phosphate solubilization, drought tolerance

## Abstract

Climate change models predict more frequent and prolonged drought events in Central Europe, which will exert extraordinary pressure on agroecosystems. One of the consequences is drought-related nutrient limitations for crops negatively affecting agricultural productivity. These effects can be mitigated by beneficial plant growth promoting rhizobacteria. In this study, we investigated the potential of cultivable bacterial species for phosphate solubilization in the rhizosphere of winter wheat at two relevant growth stages - stem elongation and grain filling stages. Rhizosphere samples were collected in the Global Change Experimental Facility in Central Germany, which comprises plots with conventional and organic farming systems under ambient and future climate. Phosphate-solubilizing bacteria were selectively isolated on Pikovskaya medium, phylogenetically classified by *16S rRNA* sequencing, and tested for *in vitro* mineral phosphate solubilization and drought tolerance using plate assays. The culture isolates were dominated by members of the genera *Phyllobacterium, Pseudomonas* and *Streptomyces.* Cultivation-derived species richness and abundance of dominant taxa, especially within the genera *Phyllobacterium* and *Pseudomonas*, as well as composition of *Pseudomonas* species were affected by wheat growth stage. *Pseudomonas* was found to be more abundant at stem elongation than at grain filling, while for *Phyllobacterium* the opposite pattern was observed. The abundance of *Streptomyces* isolates remained stable throughout the studied growth stages. The temporal shifts in the cultivable fraction of the community along with considerable P solubilization potentials of *Phyllobacterium* and *Pseudomonas* species suggest functional redundancy between and among genera at different wheat growth stages. Phosphate-solubilizing *Phyllobacterium* species were assigned to *Phyllobacterium ifriqiyense* and *Phyllobacterium sophorae.* It is the first time that phosphate solubilization potential is described for these species. Since *Phyllobacterium* species showed the highest drought tolerance along all isolates, they may play an increasingly important role in phosphate solubilization in a future dryer climate.

## Introduction

Winter wheat (*Triticum aestivum* L.) is the most commonly grown crop in Germany accounting for 54% of the total agricultural area in 2017 (Schmeling, 2017). At the same time wheat is the second most produced crop worldwide after maize (Statista, 2018). The production of winter wheat depends on frequent precipitation and sufficient nutrient supply (Fischer, 2003). Its cultivation is hence restricted to appropriate regions and is commonly supported by sufficient fertilization. Besides nitrogen and potassium, phosphorus (P) has to be applied, since the plant-available P in the rooted topsoil often represents a growth limiting factor. However, P fertilization does not necessarily increase P availability. Up to 90% of fertilized mineral P is quickly immobilized by reactive cations, such as Al^3+^ or Fe^3+^ in acidic or Ca^2+^ in calcareous or neutral soils (Oehl et al., 2002). At a global scale, models suggest an increase in P input into agricultural systems of 51–86% by the year 2050 (Mogollón et al., 2018) to maintain productivity. In parallel, the source for P fertilizer – natural rock phosphate – is expected to deplete in 50–100 years (Cordell et al., 2009). Regarding the application of organic fertilizers phosphorus is bound into complex compounds and first has to be released before plant uptake.

Processes that release P from mineral and organic compounds in the soil are mainly driven by microorganisms. Therefore, to counteract P limitation, plants enrich a variety of beneficial microbes in their rhizospheres by secreting nourishing, carbon-containing rhizodeposits. Besides mycorrhizal fungi, plant growth promoting rhizobacteria (PGPR) can enhance plant P uptake. PGPR thereby stimulate root growth, release P from immobilized inorganic and organic P pools in the soil, and transport solved P compounds to the plant’s roots (Artursson et al., 2006). However, these mutual interactions between the plant and its rhizosphere community are highly dynamic and related to the plant growth stage as well as to environmental conditions.

Seasonal shifts in rhizosphere microbiomes related to plant growth stages are well documented for canola and grasses (Dunfield and Germida, 2003; Wolsing and Priemé, 2004; Francioli et al., 2018). The changes are mainly caused by variations in the quantity and quality of carbon-derived rhizodeposits serving as energy sources for soil microbes (reviewed in Bais et al., 2006). These rhizodeposits are predominantly released into the rhizosphere when plants are actively growing (Badri and Vivanco, 2009). Thus, flowering plants (Aulakh et al., 2001; Lucas García et al., 2001) mainly secrete root exudates during stem elongation, booting and flowering stages, and much less during fruit development and ripening stages. Malhi et al. (2006) also found stem elongation and tillering to be the stages with maximum nutrient uptake of cereals. Investigations of the wheat rhizobiome often focus on the stages of tillering and flowering, but also on grain filling (predominantly in field experiments, e.g., Juhnke et al., 1987; Creus et al., 2004; Naiman et al., 2009; Ilyas and Bano, 2010) or are independent of certain growth stages throughout the first weeks of wheat development (predominantly in pot experiments, e.g., Kasim et al., 2013; Timmusk et al., 2014). Clearly, more information on the interrelationships between plant growth stages and the PGPR are needed.

Besides the seasonal dynamics, microbial communities in agricultural soils have to cope with frequent disturbances. Agricultural management practices such as fertilization, tillage, pesticide application and crop rotation including brown fallow periods cause dramatic changes of C supply patterns within the soil rooting zone. It was shown that tillage (Frey et al., 1999; Kladivko, 2001) and crop rotation (Tiemann et al., 2015) lead to a decrease in fungal biomass (Smith and Read, 2010) and diversity (Verbruggen et al., 2010). A recent study of Banerjee et al. (2019) also demonstrated that land use intensification reduces fungal network complexity in conventional and even in no tillage land use systems. In contrary, bacterial biomass was nearly unaffected (Elliott et al., 1988), leading to establishment of a primarily bacteria-based food web in managed agroecosystems (Hendrix et al., 1986; de Vries and Shade, 2013). Therefore, we focused on the bacterial rather than the fungal community in this study.

The high adaptation potential of soil bacteria to changing conditions is related to their high growth and mutation rates, as well as rapid recombination by lateral gene transfer (Badri and Vivanco, 2009). Moreover, to maintain crucial soil processes after disturbances and under changing conditions, the huge bacterial diversity allows quick and diverse reordering of the active community (Kennedy, 1999). According to Sheik et al. (2011), stress conditions such as the combination of warming and drought effects lead to less diverse microbial populations. In contrast, functions that have to be provided for plants by microbes may be even more essential under stress conditions and a more complex population structure may arise, since more complex co-occurrence networks improve complementarity and efficiency (Tardy et al., 2014; Karimi et al., 2017). Thus, soil bacterial communities play a key role in stress resistance and recovery after disturbances of agroecosystems (Shade et al., 2012).

Most field studies on beneficial wheat rhizosphere microbes and their plant growth promoting properties have been performed in arid and semiarid regions of India (Rana et al., 2011; Jog et al., 2014; Kumar et al., 2014; Singh and Lal, 2016; Verma et al., 2016), Pakistan (Ilyas and Bano, 2010; Naveed et al., 2014) and Argentina (Creus et al., 2004; Fischer et al., 2007). As these regions are strongly limited in water supply and therefore in the amount of available nutrients, which are solved in the soil water, the potential of PGPR to support wheat growth under drought conditions by different functional properties is of high interest. In contrast, studies in temperate zones with moderate precipitation are underrepresented, although climate change already causes obvious negative effects on agroecosystem productivity (Ciais et al., 2005; Trnka et al., 2014). For instance in 2018 East and Middle Germany experienced a severe drought period, which led to premature ripening and an early harvest of crops (BMEL, 2018). Drought along with nutrient limitation caused yield losses will likely increase in future as climate change models predict altered precipitation patterns with extended drought periods combined with higher temperatures in the vegetation period (Petersen and Weigel, 2015). Therefore, the role of soil bacteria and their potentials for tolerance and resilience of agroecosystems in the temperate zones is of increasing importance.

To understand the role of the rhizosphere bacterial community in providing P to wheat plants over the growing season, it is indispensable to identify P-solubilizing key species and their functionality in dependency on wheat growth stage while simultaneously considering the impacts of the two most important global change drivers, agricultural management and climate (Sala et al., 2000). In order to address this issue, we investigated P-solubilizing rhizosphere bacteria at two relevant wheat growth stages, namely stem elongation (vegetative biomass production) and grain filling stage (generative biomass production). Wheat samples were collected in the Global Change Experimental Facility (GCEF, Schädler et al., 2019). The design of the experimental field platform allows the comparison of two different farming systems (conventional vs. organic agriculture) exposed to either ambient climate, at present, or future expected climate scenario with warming and increased summer drought in the latter. Within this experimental setup we used a cultivation-dependent approach based on Pikovskaya medium (Pikovskaya, 1948) to isolate exclusively for P-solubilizing bacteria. We decided for tri-calcium phosphate (TCP) as insoluble phosphate source in the neutral Pikovskaya media since the pH in the plots of the GCEF is mainly neutral and reactive anion Ca^2+^ is predominantly binding mineral P in these soils forming stable complexes (Oehl et al., 2002, Bashan et al., 2013). Solubility of TCP increases with decreasing pH. Acidification by production and release of organic acids is thus one possibility to release phosphate from insoluble TCP source and is therefore an indicator for the potential of phosphate solubilization *in vitro* (Bashan et al., 2013). We compared the *in vitro* P solubilization potential and the drought tolerance of the predominantly isolated P-solubilizing taxa at the stem elongation stage (vegetative biomass production) with those present at the grain filling stage (generative biomass production) in different land use systems and under different climatic conditions. We aimed to distinguish the responses in terms of structural resistance and/or functional redundancy between and among cultivable P-solubilizing bacterial taxa. Under changing conditions, identical taxa and functions would suggest adaptation and resistance. In contrast, shifts in the cultivable fraction of the community would either reflect changes in functionality or reveal functional redundancy among P-solubilizing bacterial species.

We hypothesized that (i) the abundances of the predominant P-solubilizing taxa in the wheat rhizosphere vary between stages of wheat development, displaying a higher abundance, diversity and activity of P-solubilizing bacteria at the stem elongation stage due to higher resource demand by plants than at the grain filling stage (Malhi et al., 2006). Furthermore, we hypothesized that (ii) increased drought decreases diversity but induces targeted structural changes within the bacterial community, in a way that P solubilization is maintained. Finally, due to a lower availability of nutrients, we expected (iii) a lower abundance, but higher activity potentials of P-solubilizing bacteria in the organic farming system compared to the conventional farming system.

## Materials and Methods

### Experimental Platform

The Global Change Experimental Facility (GCEF) (see http://www.ufz.de/index.php?en=40038 for general information and visualizations) was established in 2013 to investigate climate change effects on managed terrestrial ecosystems (Schädler et al., 2019). The GCEF is located at the field research station of the Helmholtz-Centre for Environmental Research (UFZ) in Bad Lauchstädt, Germany (51°23′35″N 11°52′55″E, 118 m a.s.l.). The site is characterized by a temperate climate with an average temperature of 9.7°C (1993–2013) and a mean annual precipitation of 525 mm (1993–2013). The soil type is a humus- and nutrient-rich Haplic Chernozem (Altermann et al., 2005). Besides three grassland management types, a conventional and an organic farming system are part of the GCEF design. In total, the facility includes 50 large plots (24 × 16 m) that allow realistic field management. The 50 plots are arranged in ten blocks, whereby each block comprises all five land use types that are randomly distributed (Supplementary Figure S1). Five blocks are subjected to ambient climatic conditions and the other five to future climatic conditions, resulting in a split-plot experimental design (Supplementary Figure S1). Ambient climate treatment refers to the actual and non-manipulated climate in terms of precipitation and temperature at the field site. According to the projected climate change within the next 50 years for Central Germany, the future climate treatment of the GCEF consists in increasing the mean daily temperature and shifting the annual precipitation pattern. Temperature is increased passively by roofing the plots during the night. Precipitation is modulated based on the actual weather conditions, with a 20% decrease of precipitation during summer (Jun-Aug) and a 10% increase of precipitation in spring (March–May) and autumn (September–November). In this study we focused on the conventional farming (CF) and organic farming (OF) systems. The CF includes a typical regional crop rotation consisting of a sequence of winter rape, winter wheat and winter barley. In two out of 3 years, the crop cycle for OF includes winter wheat and winter barley just as for the CF. In the first and fourth year of this bipartite crop sequence, the nitrogen-fixating legumes alfalfa and white clover are included in the crop cycle, respectively. Herbicides, fungicides, insecticides and plant growth regulators are applied as usual in CF practice (Schädler et al., 2019). For OF only mechanical weed control and a restricted use of pesticides are allowed. Whereas N, P, and K in CF is applied in form of mineral fertilizers, N fertilization in OF is done by the inclusion of legumes in the crop rotation and further fertilization is restricted to the application of rock phosphate (P-Ca-Mg) and patent kali (K-Mg-S) in the first year of the crop cycle.

### Sampling and Characterization of Soil Chemical Parameters

Rhizosphere from root systems of winter wheat was collected in organic (OF) and conventional farming (CF) plots of the GCEF exposed either to ambient (A) or simulated future (F) climate conditions. From each of these plots of the GCEF (20 plots) – CF-A (5 plots), CF-F (5 plots), OF-A (5 plots) and OF-F (5 plots) – three plants were taken with root system finalizing in 15 samples per treatment. To cover relevant growth stages for vegetative and generative biomass production over the growing season, sampling campaigns took place at the 26th of May 2015 (BBCH growth stages 37–39, representing stem elongation phase) and at the 8th of July 2015 (BBCH growth stages 75–77, representing the grain filling stage), respectively. Growth stages were identified according to BBCH-scale (“Biologische Bundesanstalt, Bundessortenamt und CHemische Industrie”). Wheat plants were carefully removed from the soil, their root systems cut and shaken to discard unattached soil, and transferred in plastic bags on ice to the laboratory. Subsequently, rhizosphere soil was collected by gently, manually loosening the root-attached soil to avoid damages to the roots. To measure soil chemical properties, bulk soil samples were collected in parallel to rhizosphere samples. Six soil cores per plot were taken to a depth of 15 cm, pooled, sieved to 2 mm and manually cleaned from organic material. The obtained rhizosphere and bulk soil were stored frozen until usage.

Soil chemical properties of the bulk soil samples were analyzed as follows: gravimetric water content of fresh soil was quantified with a halogen moisture analysator (Mettler Toledo, Gießen, Deutschland). pH was measured with an electrode after shaking the soil for 1 h in 0.01 M CaCl2 (1:2.5 w/v). Amounts of total carbon and nitrogen content were determined from air-dried soil using an elemental analyzer (Elementar Vario EL III, Elementar, Hanau, Germany). For analysis of mineral nitrogen 5 g of fresh soil were suspended in 20 ml of 1 M KCl solution and measured per flow injection analysis (FlAstar 5000, Foss GmbH, Rellingen, Germany). Extraction of labile phosphorus in the soil was performed with double lactate solution (1:50 w/v) at pH 3.6 and determined using the colorimetrical molybdenum blue method (Murphy and Riley, 1962).

### Selective Isolation and Phylogenetic Classification of Phosphate Solubilizing Bacteria From Wheat Rhizosphere

For cultivation and isolation of bacteria from wheat rhizosphere, 0.5 g of fresh rhizosphere soil was suspended in 50 ml of distilled water and stirred for 5 min to break up soil particles and bring bacteria in suspension. Subsequently, the samples were sonicated for 10 s and stirred for another 2 min. Mineral tri-calcium phosphate solubilizing bacteria were isolated from a 1:200 (v/v) dilution of this rhizosphere soil suspension by plating 50 μl of it in triplicate on Pikovskaya agar (Pikovskaya, 1948), supplemented with the fungicide cycloheximide (10 mg/ml). After 2 weeks of incubation at 25°C, colony forming units (CFU) were counted. Bacterial strains produce and release organic acids to solubilize P from insoluble tri-calcium phosphate source, therefore, forming a clear zone in the otherwise turbid agar around the colony - from heron referred to as halo. Up to 25 colonies (Supplementary Table S4) with the largest halos were collected for each sample from the Pikovskaya plates and transferred to yeast malt extract (YME) agar plates. To identify and store these isolates, polyethylene glycol (PEG) and glycerol stocks were prepared and frozen. Cells in the PEG stock were destroyed mechanically with glass beads by vortexing to release their DNA into the liquid. Polymerase chain reaction (PCR) was performed on the DNA obtained from the PEG stocks with the primers 27f (10 μM – 5′-AGAGTTTGATCMTGGCTCAG-3′: Lane, 1991) and 1492r (10 μM – 5′-GGTTACCTTGTTACGACTT-3′: Lane, 1991) and Promega Green (Promega, Madison, WI, United States). Partial *16S rRNA* sequencing was performed with the primer BAC 341f primer (10 μM – 5′-CCTACGGGAGGCAGCAG-3′: Muyzer et al., 1993) using Big Dye Termination Mix (GeneCust Europe, Dudelange, Luxemburg). Quality control of the sequences was done manually using Sequencher 5.4.5. Quality-checked sequences were compared with type strain reference sequences of the National Center of Biotechnology Information (NCBI), and subsequently clustered according to a level of 99.5% identity. Representative sequences of each cluster were aligned using neighbor-joining algorithms of the MAFFT server (Katoh and Standley, 2013). Phylogenetic trees were constructed and visualized along with the trait and abundance data using Evolview (Zhang H. et al., 2012). All *16S rRNA* gene sequences were deposited in the NCBI database with continuous accession numbers from MK637853 to MK638668 (Supplementary Table S1).

### Estimating the Functional Properties of Isolated Bacteria

Pure cultures of the dominant P-solubilizing taxa were characterized by functional tests using agar plate bioassays. For each isolate, organic acid production to solubilize tri-calcium phosphate was quantitatively and qualitatively determined on Pikovskaya agar (Pikovskaya, 1948) by the formation of a clear zone in the otherwise turbid agar around the colony. Drought tolerance of the isolates was determined by comparing their growth on YME to surface polyethylene glycol 6000 (PEG) infiltrated YME. Thus, in this study drought tolerance potential of the strains was not related to the formation of dormant spores, but to their potential to maintain metabolisms and growth under water limiting conditions. To simulate severe drought stress, PEG concentration was set to 500 g/L, which corresponds to an osmotic potential level of around −1.1 MPa (Verslues et al., 2006).

For both bioassays, the strains were grown to the end of the exponential growth phase in liquid YME and 1 μl of each liquid pre-culture was applied on the test media in triplicates. P solubilization was assessed after 14 and drought tolerance after 3 days of incubation in the dark at 25°C. Phosphate solubilization was quantified as P release derived from phosphate concentration per area of plate and valued using two complementary indices. On the one hand, P release was calculated based on the whole area including colony plus halo, representing the total amount of P released by the colony (Phosphate Solubilization Index 1, PSI 1). Since primarily the outer cells of a colony determine the distance between colony rim and halo, we further calculated P release (Phosphate Solubilization Index 2, PSI 2) based solely on the halo around the colony (halo diameter = twice the distance from colony rim to the end of the halo), representing cell-specific, rather than colony-specific P solubilization potential. Drought stress tolerance was quantified based on the percentage differences of colony diameters on PEG-YME agar, compared to control YME agar. For statistical analysis only data from bacterial strains growing on the functional media were used.

### Statistical Analyses

All statistical analyses were performed in the open source program R version 3.4.0 (2017-04-21), GNU project (R Core Team, 2017). An analysis of variance (ANOVA) was run to evaluate, if the abundance and traits of P-solubilizing bacteria were influenced by climate, land use, wheat growth stage, or interaction of these factors. The ANOVA was followed by a multiple comparison analysis with Tukey HSD to identify respective differences in the means of groups. To test for differences in phosphate solubilization potentials and drought tolerances between dominant bacterial taxa, the factor taxa was added to the formula resulting in a four factorial ANOVA. Significance levels were defined according to ***p < 0.001, **p < 0.01, and *p < 0.05.

To test for homogeneity of multivariate dispersion of *Phyllobacterium*, *Pseudomonas* and *Streptomyces* phylogenetic clusters among and between test factors climate, land use and wheat growth stage, betadisper function (R, vegan package) was run based on a dissimilarity matrix using relative abundance (Bray Curtis) and incidence data (Jaccard). Biodiversity indices, i.e., species richness and Shannon-Weaver-index were calculated to test for impacts of climate, land use and wheat growth stage on structure and composition of cultured *Phyllobacterium*, *Pseudomonas* and *Streptomyces* species. ANOVA was applied to test for differences in cluster dispersion regarding structure and composition of *Phyllobacterium*, *Pseudomonas* and *Streptomyces* species between and among test factors.

## Results

### Soil Chemical Parameters

Soil chemical properties of the studied samples are summarized in Supplementary Table S2. Since our study was performed only 2 years after establishment of the GCEF, basic soil parameters did not differ between treatments and showed comparable values of pH (6.7), total organic carbon (1.9%) and total nitrogen (0.16%). Available P and mineral N were higher in CF than in OF, but the difference was only significant for mineral N (Supplementary Table S2). The soil moisture differed between the ambient and future plots at the days of sampling and showed average values of 10.6 and 15.9% at stem elongation stage in May and at grain filling stage in July, respectively (Supplementary Table S2).

### Effects of Wheat Growth Stage, Land Use and Climate on P-Solubilizing Rhizosphere Bacteria

Colony counts on Pikovskaya agar indicated higher numbers of P-solubilizing rhizobacteria at the stage of grain filling in July compared to the stage of stem elongation in May (*p* < 0.001). At both growth stages more bacteria were detected in CF than in OF (May: *p* = 0.008, July: *p* = 0.03) and under ambient climate than under future climate at grain filling stage in July (*p* = 0.02) (Supplementary Figure S2).

**FIGURE 1:**
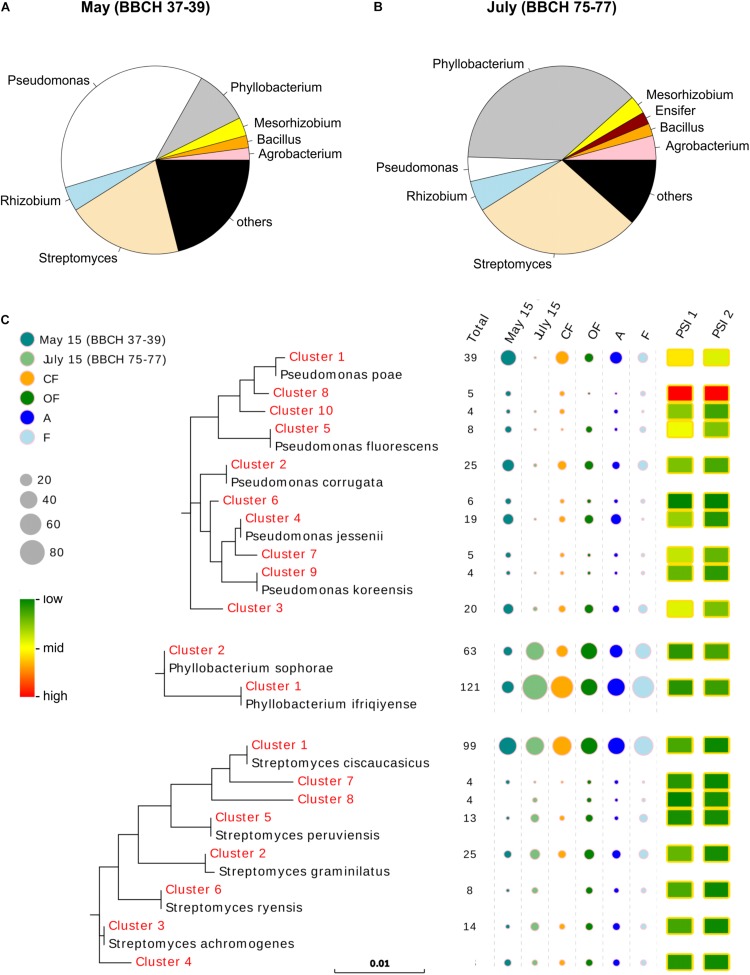
Identity, abundance and activity levels of cultivable mineral phosphate-solubilizing bacteria in the wheat rhizospheres. Taxa have been summarized on genus level and the most dominant ones, with a relative abundance ≥2% within the collection, are shown in the pie charts at **(A)** stem elongation stage in May 2015 and **(B)** grain filling stage in July 2015. The predominantly isolated taxa of the genus *Phyllobacterium*, *Pseudomonas* and *Streptomyces* were clustered (identity level of 99.5%) and **(C)** phylogenetic trees were created for dominant clusters (number of isolates >3) with reference sequences from NCBI. Information on abundance of the dominant clusters within treatment levels of growth stage (stem elongation in May, grain filling in July), climate (A-ambient, F-future) and land use (CF-conventional farming, OF-organic farming) are given as circles, whereas circle size is related to the number of isolates. Moreover, the average phosphate solubilizing potentials for each bacterial *16S rRNA* gene cluster were calculated for PSI 1 and PSI 2 and differences in expression levels are indicated using a heatmap.

In total 410 bacterial isolates were obtained from samples collected at the stage of stem elongation and another 407 strains from samples at the grain filling stage (Supplementary Table S4). Within the isolates collected from the May samples at stem elongation stage, the dominant taxa (relative abundance ≥2%) belonged to the genera *Pseudomonas* (150 isolates = 37%), *Streptomyces* (81 isolates = 20%), *Phyllobacterium* (37 isolates = 9%), *Rhizobium* (20 isolates = 5%), *Mesorhizobium* (16 isolates = 4%), *Bacillus* (12 isolates = 3%) and *Agrobacterium* (10 isolates = 2%), while in the collection from July samples at the grain filling stage *Phyllobacterium* (147 isolates = 36%), *Streptomyces* (116 isolates = 29%), *Rhizobium* (24 isolates = 6%), *Agrobacterium* (20 isolates = 5%), *Pseudomonas* (17 isolates = 4%), *Mesorhizobium* (16 isolates = 4%), *Ensifer* (10 isolates = 2%) and *Bacillus* (9 isolates = 2%) (Supplementary Table S5) were the dominant genera. Thus, along both wheat growth stages, the taxa belonging to the genera *Phyllobacterium*, *Pseudomonas* and *Streptomyces* were found to dominate the bacterial collections, jointly accounting for 65% and 69% of the total isolates at stem elongation in May and grain filling stage in July, respectively. However, between the two plant growth stages, shifts in abundances of these dominant taxa were observed (Figures 1A,B and Supplementary Table S5). At the stage of stem elongation in May, *Pseudomonas* were predominant with 150 isolates accounting for more than one third of the bacterial isolates, whereas *Phyllobacterium* species only made up a small fraction with 37 isolates (9%). Conversely, at the grain filling stage in July, a much higher abundance of P-solubilizing *Phyllobacterium* species (147 isolates = 36%) than *Pseudomonas* species (17 isolates = 4%) was detected. Contrary to these, the abundance of *Streptomyces* isolates was less affected by the wheat growth stage, when 81 isolates (20%) and 116 isolates (29%) were obtained from stem elongation stage and grain filling stage, respectively.

Comparable abundances along the two growth stages were also found for the less abundant taxa of the genus *Agrobacterium*, *Bacillus* and *Rhizobium*, while the genus *Ensifer* was predominantly detected in July (Figure 1B and Supplementary Table S5). Some of the less abundant genera also showed indications for land use and climate-specific patterns. *Agrobacterium* and *Buttiauxiella* were more frequently detected in CF than in OF, whereby *Agrobacterium* spp. were mainly isolated from ambient climate plots, while *Buttiauxiella* spp. were exclusively detected in future climate plots (Supplementary Table S5). A comparable enrichment under future climatic conditions was also observed for *Bacillus*, and species were more often isolated from OF than from CF (Supplementary Table S5).

To assess species identity and trait distribution within the dominant genera, we clustered the *Phyllobacterium*, *Pseudomonas* and *Streptomyces* isolates according to their *16S rRNA* gene sequences at a similarity level of 99.5%. Phylogenetic trees were constructed with representative sequences of clusters comprising more than three isolates (Figure 1C), resulting in two *16S rRNA* gene clusters for *Phyllobacterium*, ten for *Pseudomonas* and eight for *Streptomyces*. The clusters of *Phyllobacterium* species were related to *Phyllobacterium ifriqiyense* (cluster 1) and *Phyllobacterium sophorae* (cluster 2). Besides the strong impact of growth stage and the fact that more isolates were assigned to cluster one than to cluster two, the abundances of the *Phyllobacterium* clusters showed divergent patterns between the two farming systems. Cluster one was more abundant in CF than in OF, while cluster two showed a contrary trend (Figure 1C and Supplementary Table S6). Phylogenetic cluster distributions of the taxa within the genus *Pseudomonas* and *Streptomyces* were more heterogeneous. *Pseudomonas* clusters grouped with *Pseudomonas poae* (clusters 1, 8, and 10), *Pseudomonas corrugate* (cluster 2), *Pseudomonas jessenii* (cluster 4 and 7), *Pseudomonas fluorescens* (cluster 5), and *Pseudomonas koreensis* (cluster 9) (Figure 1C). *Streptomyces* cluster 1 contained the highest number of isolates and grouped with clusters 7 and 8, and reference sequence *Streptomyces ciscaucasicus* (Figure 1C). The other less abundant *Streptomyces* clusters grouped with reference sequences of *Streptomyces graminilatus* (cluster 2), *Streptomyces achromogenes* (clusters 3 and 4), *Streptomyces peruviensis* (cluster 5), and *Streptomyces ryensis* (cluster 6) (Figure 1C). Effects of climate and land use treatments on *Streptomyces* clusters could not be detected, but for *Pseudomonas* cluster 1. Representatives of this cluster were approximately twice as often isolated from CF and plots with ambient climatic conditions compared to OF and plots with future climatic conditions (Supplementary Table S6).

**FIGURE 2:**
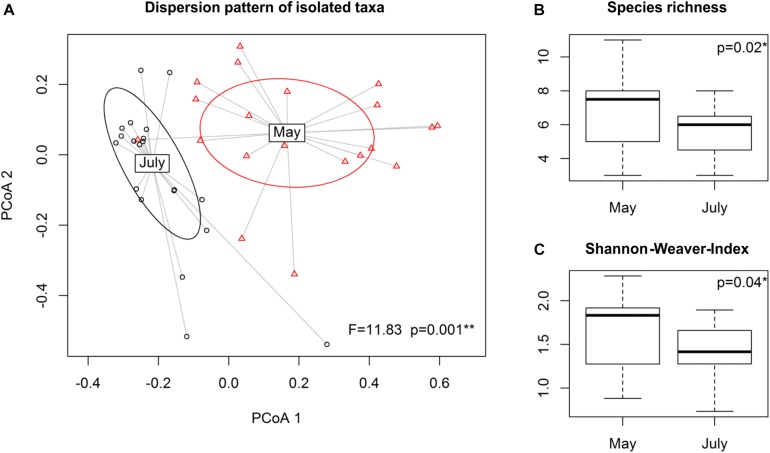
Effect of time on composition of isolated rhizobacterial taxa among and within the dominating genera *Phyllobacterium*, *Pseudomonas* and *Streptomyces*. Running betadisper function based on Bray-Curtis dissimilarity **(A)** dispersion patterns of the isolated taxa are presented in an ordination plot. Diversity indices **(B)** species richness and **(C)** Shannon-Weaver-Index were calculated, followed by ANOVA, testing for differences in isolated species composition of the three dominant genera between the two sampling points, May (stem elongation stage, BBCH 37-39) and July (grain filling stage, BBCH 75-77) 2015, respectively.

Dispersion patterns for *Phyllobacterium, Pseudomonas* and *Streptomyces* clusters significantly changed over the growing season (*p* = 0.001) (Figure 2A). At the genus level, compositional shifts were observed among the taxa of the genus *Pseudomonas* (*p* = 0.006) and *Phyllobacterium* (*p* = 0.04), but not for *Streptomyces* (Supplementary Figure S3). At cluster level, a shift in the cultivable fraction of the community was detected only for clusters of genus *Pseudomonas* (*p* = 0.02). Species richness (Figure 2B) and Shannon-Weaver diversity index (Figure 2C) were found to be higher at stem elongation stage of May samples compared to grain filling stage of July samples with *p* = 0.02 and *p* = 0.04, respectively. Effects of climate or land use on dispersion patterns and diversity indices could not be observed.

### P Solubilization Potentials of Key Taxa

**FIGURE 3:**
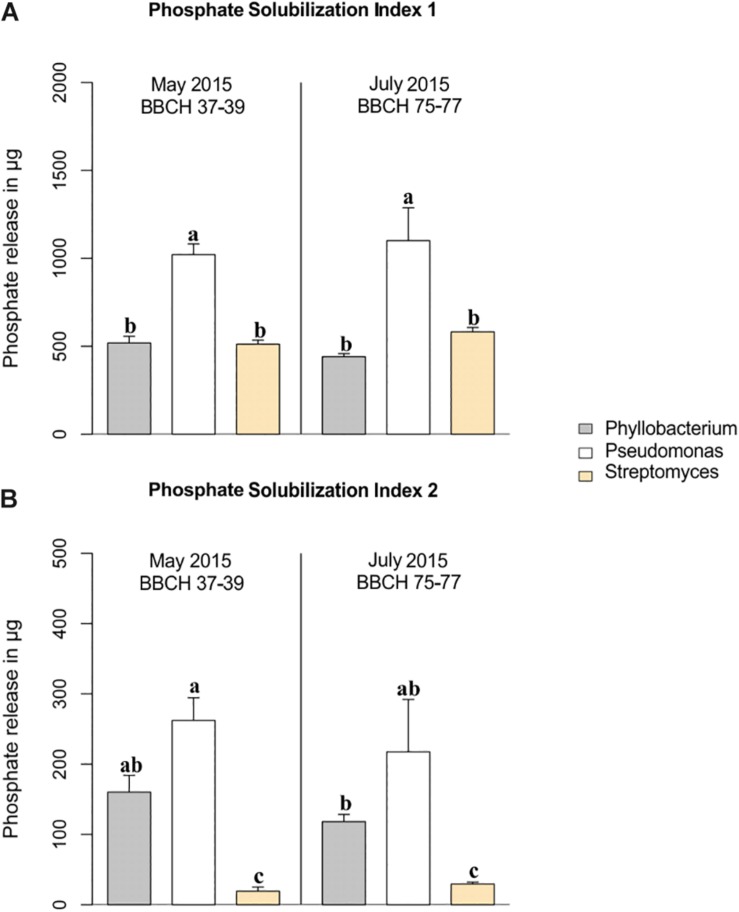
Phosphate-solubilizing potentials of the three dominant genera. Comparison of the two indices, **(A)** PSI 1 and **(B)** PSI 2 for *Phyllobacterium*, *Pseudomonas* und *Streptomyces* isolates at stem elongation in May and at grain filling in July 2015 sampled from wheat rhizosphere. Standard errors are given in the bars. Significant differences were calculated for the both indices separately and are marked by different letters (ANOVA and Tukey HSD Test).

Phosphate-solubilizing potentials of dominant taxa within the genera *Phyllobacterium*, *Pseudomonas*, and *Streptomyces* isolated from the wheat rhizospheres at the stem elongation stage in May, as well as at the grain filling stage in July are presented for PSI 1 and PSI 2 in Figures 3A,B, respectively. Among the taxa of the three genera, *Pseudomonas* species expressed highest P solubilization potentials irrespective of employed indices, PSI 1 (Figure 3A) and PSI 2 (Figure 3B), and at the two growth stages studied. *Phyllobacterium* species showed approximately half of the P solubilization potential of the *Pseudomonas* species at both growth stages independently of the used P solubilization index. In contrast, the assessment of the P solubilization potentials of *Streptomyces* species depended on the index used. Excluding the colony size for the assessment of P solubilization, as done in PSI 2, clearly revealed significantly lower P solubilization potentials of *Streptomyces* species compared to taxa of the two other genera (*p* < 0.001) (Figure 3B).

Regarding the effect of studied impact factors within the three dominant genera, neither plant growth stage (Figure 3), nor climate or land use affected the P solubilization potential of *Phyllobacterium* and *Streptomyces*. However, interaction effects of climate and land use were found for *Pseudomonas* species, which showed higher P solubilization potentials under future climatic conditions compared to ambient climatic conditions in CF (*p* < 0.001 for PSI 2) (Supplementary Figure S4). Furthermore, under future climatic conditions, P solubilization potentials of *Pseudomonas* species were significantly higher in CF compared to OF (*p* < 0.001 for both indices, Supplementary Figure S4).

The phylogenetic cluster-specific, mean P solubilization potentials of the genera *Phyllobacterium* and *Streptomyces* revealed consistent P solubilization potentials in either employed index (Figure 1C and Supplementary Table S6). In contrast to that, the average P solubilization potentials of the individual clusters assigned to the genus *Pseudomonas* showed a huge variance (Figure 1C and Supplementary Table S6). While, *Pseudomonas* cluster 6 showed a low P solubilization potential, the isolates assigned to cluster 8 expressed the highest average P solubilization potential among all clusters of all three dominant genera. Even more important, the most abundant *Pseudomonas* cluster (cluster 1, represented by 39 isolates = 23% of all *Pseudomonas* isolates) showed a high average P solubilization potential which exceeded mean P solubilization of all genera and clusters, besides the aforementioned *Pseudomonas* cluster 8 (Figure 1C and Supplementary Table S6).

### Drought Tolerance of Key Taxa

Drought stress tolerance was quantified as relative difference in colony size on PEG medium compared to control medium. Thus, higher drought tolerance was reflected by higher maintenance of colony size (maximum 100%) and *vice versa*. *Pseudomonas* species (35.1% at stem elongation in May and 35.7% at grain filling stage in July) and *Streptomyces* species (43.3% in May and 46.7% in July) were less resistant to drought compared to species assigned to the genus *Phyllobacterium* (54.1% in May and 63.6% in July) (Table 1). Although the low number of isolates at stem elongation in May samples prevents from drawing significant conclusions, species drought tolerance of genus *Phyllobacterium* was improved in the course of the growing season (Table 1). Total drought tolerance of taxa belonging to the genera *Pseudomonas* and *Streptomyces* did not change throughout the observed growth stages, but show different behavior under ambient and future climatic conditions. Under ambient climate an increase and under future climate conditions a decrease of drought tolerance could be observed for *Pseudomonas* species, while for *Streptomyces* species the opposite pattern showed up (Table 1). Land use did not impact key genera’s drought tolerance, but the climate treatment affected average drought tolerance of the genus *Streptomyces*. Strains isolated from plots with future climatic conditions showed higher drought tolerance (52.4%) as compared to those isolated from plots with ambient climate (38.6%) (*p* = 0.01). This trend was also observed at the cluster level, even though it was not significant due to the low number of isolates per cluster (Supplementary Figure S5). In contrast to streptomycetes, climate treatment showed no effect on the drought tolerance of taxa within the genera *Phyllobacterium* and *Pseudomonas*.

**TABLE 1:** Drought resistance of the members of three dominant P-solubilizing genera.

	***Phyllobacterium***		***Pseudomonas***		***Streptomyces***	
	**May**	**July**	**May**	**July**	**May**	**July**
	**BBCH**	**BBCH**	**BBCH**	**BBCH**	**BBCH**	**BBCH**
	**37–39**	**75–77**	**37–39**	**75–77**	**37–39**	**75–77**
**Ambient**	55.9% **ab**	62.5% **a**	34.2% **b**	55.2% **ab**	39.6% **b**	37.8% **b**
	(6.4)	(3.1)	(3.9)	(15.2)	(4.8)	(4.1)
**Future**	53.2% **ab**	64.4% **a**	36.2% **b**	20.2% **b**	47.8% **ab**	54.9% **a**
	(4.0)	(2.5)	(3.9)	(16.3)	(6.1)	(4.7)
**Total**	54.1% **de**	63.6% **d**	35.1% **f**	35.7% **ef**	43.3% **ef**	46.7% **e**
	(3.4)	(2.0)	(2.7)	(12.3)	(3.8)	(3.2)

*Mean levels of drought resistance exhibited by the bacterial isolates from grain filling stage (BBCH 37-39) in May and stem elongation stage (BBCH 75-77) in July under ambient and future climatic conditions. Standard error is given in parenthesis. Significant differences according to Tukey post hoc test are marked by different letters for ambient/future (a–b) and total (d–f), respectively.*

At the cluster level, a marginal higher drought tolerance of the species isolated from plots with future climatic conditions, compared to those isolated from ambient plots was observed for *Phyllobacterium* cluster 2 (Supplementary Figure S5). Within the genus *Pseudomonas*, we observed no increase in drought tolerance of species from future climate plots except for the low abundant cluster 10 and, to a lower extent, for cluster 3 and cluster 8 (Supplementary Figure S5). Overall, species of both *Phyllobacterium* clusters exhibit a significantly higher drought tolerance as compared to those of the predominant clusters of *Pseudomonas* (clusters 1 to 4) to and *Streptomyces* species (clusters 1 to 4) (Supplementary Figure S5 and Supplementary Table S6).

## Discussion

In this study we have shown that members of the genera *Phyllobacterium, Pseudomonas* and *Streptomyces* are predominant within the cultivable fraction of the P-solubilizing community in the wheat rhizosphere along both growth stages studied, namely vegetative biomass production (BBCH 37–39, stem elongation) and generative biomass production stages (BBCH 75–77, grain filling). All taxa within the three dominant genera showed high resilience against differences in studied agricultural management practices and climatic conditions, but structure shifted with wheat growth stages. This shift was mainly driven by changes among P-solubilizing taxa of the genera *Pseudomonas* and *Phyllobacterium*, as well as on single taxa level within the genus *Pseudomonas*. The considerable *in vitro* P solubilization potential of both genera *Pseudomonas* and *Phyllobacterium* pleads for functional redundancy upon structural changes within the cultivable fraction of the P-solubilizing community. While the genera *Pseudomonas* and *Streptomyces* comprise well-known P solubilizers, *Phyllobacterium* species have rarely been associated with P solubilization. Considering their medium-borne P solubilization potential in combination with an accentuated tolerance of actively growing colonies to drought conditions, *Phyllobacterium* species may play an important role in P supply for plants under drought.

### Wheat Growth Stage and Farming System Drive Abundance and Identity of Phosphate-Solubilizing Rhizobacteria

The abundance of cultivable P-solubilizing rhizobacteria (PSR), mirrored by CFU per gram dry soil, was higher at the grain filling stage of wheat in July than at the stem elongation stage in May and contradicts our assumptions made in the first hypothesis. This discrepancy might be related to higher soil moisture at the day of sampling in July, since increased water availability supports microbial growth and enhances metabolic activities (Williams and Rice, 2007). Due to the realistic scenario-based approach of the GCEF, the soil moisture is mainly dependent on the actual weather. However, the impact of the climate treatment was clearly evident for the annual soil moisture dynamics of 2015 (Schädler et al., 2019) and led to significantly reduced winter wheat yields under future climatic conditions compared to ambient ones (Supplementary Table S3).

According to our third hypothesis, the conventional farming (CF) showed higher PSR numbers than the organic farming (OF), which is likely related to the higher nutrient availability in CF. Physicochemical soil parameters are well known as important drivers of microbial biomass (Widmer et al., 2006) as well as of the community structure and composition (Cleveland and Liptzin, 2007; Lauber et al., 2008; Francioli et al., 2016). Nevertheless, we consistently identified *Phyllobacterium*, *Pseudomonas* and *Streptomyces* species as the predominant cultivable PSR in both farming systems. In general, plants recruit beneficial PGPR from the surrounding soil to their rhizospheres (Badri and Vivanco, 2009) by plant specific rhizodeposition patterns (Paterson et al., 2007). This selection effect may mask environmental impacts on the community structure and composition in the rhizosphere and, thus, explain the low differences in the identity of predominant PSR isolated across both farming systems.

### Phosphate-Solubilizing Potential First Time Reported for *P. ifriqiyense* and *P. sophorae*

Multiple studies report *Streptomyces* (Molla et al., 1984; Jog et al., 2014) and *Pseudomonas* species to be strong P solubilizers (e.g., Richardson et al., 2009; Marasco et al., 2012; Sharma et al., 2013) and successful colonizers of the wheat rhizosphere (Mohammadi, 2012; Jog et al., 2014). Conversely, members of the genus *Phyllobacterium*, taxonomically classified to the order Rhizobiales, are hardly described for their P solubilization potential, but rather for their biocontrol activities (Lambert et al., 1990; Aisyah et al., 2017) or the capacity to fix nitrogen (Rojas et al., 2001). Our study now provides evidence for the potential of two *Phyllobacterium* species, namely *P. ifriqiyense* and *P. sophorae*, to *in vitro* solubilize P from its bound mineral form in the rhizosphere of winter wheat in the temperate climatic zone. The two strains have first been isolated from root nodules of *Astragalus algerianus* and *Lathyrus numidicus* in South Tunisia *- P. ifriqiyense* - (Mantelin et al., 2006), and of *Sophora flavescens* in China *- P. sophorae* - (Jiao et al., 2015). Only two further previous studies indicate the potential of *Phyllobacterium myrsinacearum* for P solubilization in subtropical soils (Chen et al., 2006) and in metal-polluted soils (Ma et al., 2013). Genome shotgun sequencing of *Phyllobacterium* sp. (National Center of Biotechnology Information, NCBI) revealed quinoprotein glucose dehydrogenase coding genes. These genes are involved in gluconic acid production and, thus, suggest a potential for mineral phosphate solubilization (Rodríguez and Fraga, 1999). This coding gene has also been indicated for *Pseudomonas* species (Miller et al., 2010) and *Streptomyces* species (Jog et al., 2014) and may, therefore, serve as indicator for P solubilization potential of Phyllobacteria *in vivo*. Therefore, the contribution of *Phyllobacterium* species to phosphate solubilization in soils and rhizospheres might be still underestimated.

### Complementarity Between *Phyllobacterium* and *Pseudomonas* at Different Wheat Growth Stages

Abundances of P-solubilizing *Phyllobacterium* and *Pseudomonas* isolates were complementary between the two wheat growth stages. Since an isolation-based approach was used, this complementarity mirrors different proportions of active microorganisms. Blagodatskaya and Kuzyakov (2013) suggested that more than 95% of the total microbial biomass are inactive at a given time point. Accordingly, active growth and dormancy phases of rhizosphere species are dependent on nutrient levels, interspecific competition as well as on the plant growth stage and its related rhizodeposition (Blagodatskaya and Kuzyakov, 2013). Plant nutrient uptake and root exudation were reported to be high at stem elongation stage and considerably lower at grain filling (Aulakh et al., 2001; Malhi et al., 2006). Of note, *Pseudomonas* species were predominantly isolated from the wheat rhizosphere at the stage of stem elongation in May. Members of this genus are well characterized for their rapid growth, high chemotactic activity and motility toward root exudates (Scher et al., 1988; Walsh et al., 2001), as well as the production of secondary metabolites (Haas and Defago, 2005) and secretion of siderophores (Kloepper et al., 1980), to metabolize rhizodeposits and outcompete other microorganisms (Paulsen et al., 2005). Indeed, root colonization of active *Phyllobacterium* species may be inhibited by pseudomonads competing for root exudates at the stage of high biomass production in May. Even though *Phyllobacterium* species are described to live in a large variety of habitats and to “communicate” with plant tissues, the group is also known to be non-pathogenic (Mantelin et al., 2006). When plants reach their mature state, the release of root exudates and competition between microorganisms in the rhizosphere decreases drastically (Hamlen et al., 1972), likely explaining the decline in abundance of *Pseudomonas* and the enrichment of *Phyllobacterium* species at the grain filling stage. Antagonistic effects have not been tested within this study, but may provide important information on colonization success in plant rhizospheres at different growth stages.

### Resistance and Functional Redundancy in Terms of P Solubilization Between and Among Dominant Phosphate Solubilizing Genera

Besides abundance shifts, the cluster dispersion patterns of the cultivated dominant taxa varied significantly between the two time points with increased species richness at the stage of stem elongation in May samples. This partly supports our first hypothesis, since the diversity decrease in July is in line with reduced carbon and nutrient availabilities (Hamlen et al., 1972). The shifts within the cultivable fraction of the PSR community were based on two different responses: changes in the dominant PSR taxa, which are mainly driven by shifts within the genera *Phyllobacterium* and *Pseudomonas*, as well as altered species composition of *Pseudomonas* species. Changes in the structure and identity of *Pseudomonas* species at different wheat growth stages indicate that upon a change in the environmental conditions, the culture derived *Pseudomonas* community is dynamic. Concurrent changes in community structure and composition according to plants growth stages are widely observed in ecology (Dunfield and Germida, 2003; Wolsing and Priemé, 2004; Francioli et al., 2018). As we found moderate to high average P solubilization potentials of taxa classified to the genera *Pseudomonas* and *Phyllobacterium* at the two growth stages studied, the observed shifts plead for functional redundancy (Allison and Martiny, 2008) between and among genera at different wheat growth stages.

Besides the effect of different growth stages over the growing season, an effect of farming system was found. Increased P solubilization potentials for *Pseudomonas* isolates from CF, compared to OF, were detected under future climatic conditions. The mechanisms behind this observation cannot be revealed within this study, but we assume soil nutrient availability to play a role in the activity of PSR under drought. Interestingly, this finding is, however, supported by a previous study performed in grasslands, where we found higher P solubilization potentials of *Pseudomonas* species under low soil moisture conditions (Breitkreuz, unpublished). Within this study, we found comparable P solubilization potentials expressed by the predominant taxa along both time points, which is in contrast to our assumptions made in hypothesis one that P solubilization potential is higher at the stem elongation stage. Higher activities of *Pseudomonas* species in CF under future climate contradicts hypothesis three expecting higher P solubilization potential in OF than in CF.

The abundances of isolated *Streptomyces* species, but also the structure and composition within the genus were only slightly affected by the wheat growth stage and not at all by the climate or land use treatments. Despite their high abundance, P solubilization potential expressed according to PSI 2 were significantly less pronounced compared to those of the isolated *Phyllobacterium* and *Pseudomonas* species. However, Battini et al. (2017) recently emphasized the capability of *Streptomyces* species to facilitate P uptake into fungal hyphae in root free compartments, which is subsequently transported via the mycorrhizosphere to maize plants. In fact, Tarkka et al. (in preparation) isolated phosphate solubilizing bacteria from bulk soils of conventional and organic farming plots of the GCEF and identified *Streptomyces* species among the most abundant phosphate solubilizes. Their lower phosphate solubilizing potentials may be caused by slower development rates and delayed activity expression, compared to the mainly fast growing *Phyllobacterium* and *Pseudomonas* species (Garrity et al., 2007). The slow growth of streptomycetes is related to the formation of complex structures, e.g., branching hyphal filaments and spores (Chater et al., 2010). Dormant spores in the soil are beneficial to endure severe drought events waiting for more favorable conditions (Viaene et al., 2016). In line with this, recently published studies observed accumulation of actinobacteria in drought-treated soils and rhizospheres of different plants (Bouskill et al., 2016; Taketani et al., 2017).

For estimating the strain-specific P solubilization potentials, two complementary indices were calculated. PSI 1, which is based on the total colony and halo size, is directly related to the total amount of released P, but the values are strongly biased by the respective colony size. Consequently, identical distances between halo and colony rim along with larger colony sizes may overrate the activity potential since bigger colonies have higher area values and vice versa. The same problem occurs when using the commonly published ratios, halo to colony size (e.g., Nguyen et al., 1992; Kumar and Narula, 1999; Pérez et al., 2007). However, the diffusion expanse of organic acids in the surrounding of bacterial colonies to solubilize bound P is mainly determined by the outermost cells of the colony. PSI 2 remedies the strong impact of the colony size that, in our case for *Streptomyces* species, may impact appraisal of the P solubilization potential. We thus suggest to focus on approaches based on diffusion expanse (i.e., the distance between halo and colony) for estimation of phosphate solubilizing potential when using plate bioassays.

### Drought Tolerance of P-Solubilizing Bacteria

The tolerance of the predominant taxa against severe drought stress was quantified in this study by water deficit bioassays with polyethylene glycol 6000 (PEG). Using this approach, we focused on whether isolated strains remained physiologically active under low moisture conditions. In this respect, evaluating growth on nutrient agar with PEG may provide information on active PSR under drought conditions.

As assumed in our second hypothesis, we consistently observed a (by trend) increased drought tolerance of the dominant taxa isolated at stage of grain filling from July as compared to those at stem elongation from May. This indicates enhanced adaptive bacterial responses against dehydration stress in summer. While we detected no land use effect on drought tolerance, we found a higher drought tolerance for *Streptomyces* species, when isolated from plots under future climatic conditions, compared to those from ambient climate plots. Overall, we observed the highest drought tolerance for *Phyllobacterium* species, followed by *Streptomyces* and consecutively *Pseudomonas* species. Soil microorganisms tolerate water stress in a variable extent (Manzoni et al., 2012). As a most common mitigation strategy, bacteria as well as eukaryotes accumulate low molecular weight compounds, so called osmolytes, once water potentials decrease (Kempf and Bremer, 1998). These compounds do not interfere with cellular functions, but stabilize cellular structures and prevent desiccation. Their production and accumulation in the abundant clusters observed within this study is established, for *Phyllobacterium* (Fujihara, 2008; Sagot et al., 2010; Zaprasis et al., 2015), but also for *Pseudomonas* species, when isolated from arid or semi-arid regions of India (Sandhya et al., 2009). However, Lennon et al. (2012) performed a study in (MI, United States, humid region) and found with decreasing water potentials that respiration of *Pseudomonas* species drastically dropped, which supports the findings for *Pseudomonas* in our study.

### Field Application Potential of P-Solubilizing Bacteria

Besides rhizosphere competence, the trait combination of high P solubilization potential and drought tolerance is a prerequisite for field applications. Inoculation with microbial species of the genera *Bacillus* and *Paenibacillus* (Zhang J. et al., 2012; Kasim et al., 2013; Kumar et al., 2014; Timmusk et al., 2014), as well as *Azospirillum* and *Pseudomonas* (Creus et al., 2004; Naiman et al., 2009; Ilyas and Bano, 2010; Kasim et al., 2013) and *Streptomyces* (Jog et al., 2014) was found to increase wheat yields in field and pot experiments. In particular, *Pseudomonas* species are well investigated, as the genus comprises species with promising plant growth promoting properties (Burr et al., 1978; Naiman et al., 2009; Rajkumar et al., 2010). In contrast, prior to this study little was known about P-solubilizing *Phyllobacterium* species, or their drought tolerance in wheat rhizosphere. They do act as PGPR e.g., in canola plants (Bertrand et al., 2001), but underlying mechanisms remain unclear. Model studies with Arabidopsis thaliana and *Phyllobacterium brassicacearum* indicated higher drought tolerance (Bresson et al., 2014). In the same context, Kechid et al. (2013) observed a delay in reproduction and lowered transpiration rate of Arabidopsis thaliana upon inoculation with *Phyllobacterium brassicacearum* accompanied by stimulated lateral root and root hair growth. Within this study we could prove *in vitro* potential for P-solubilization of two taxa in the genus *Phyllobacterium*, which has to be further justified *in vivo* under different biotic and abiotic conditions. To check applicability of these species as phosphate delivering biofertilizers, the timing of inoculation and bacterial level has to be defined in pretests to ensure survival of the inoculated strains, but also to keep viability of the seeds. Bashan (1986) found inoculation at early growth stages of wheat with 10^5^–10^6^ colony forming units per ml to be optimal for *Azospirillum* and *Pseudomonas* strains to increase productivity. The success of colonization depends on different factors and is favored by high mobility of the bacteria along the growing root, rapid multiplication and the ability to colonize the rhizoplane and inner root tissues (Höflich et al., 1995). Therefore, the colonization potential for different plant root systems, the effect of different soil parameters and antagonistic effects within the native microbial community has to be quantified in inoculation experiments under natural (non-sterile) and controlled (sterile) conditions (Gaskins et al., 1985; Höflich et al., 1995). Finally, the strains have to be tested for their plant growth promoting activities exploiting various sources of insoluble P in the soil, besides tri-calcium phosphate also other insoluble inorganic and organic phosphate sources (Bashan et al., 2013), and their drought stress tolerance under natural conditions in pot and field experiments. Nevertheless, the non-pathogenic status and high plant-interaction potential of *Phyllobacterium* species, along with the considerable P solubilization potential *in vitro* and high drought tolerance revealed in this study, make this group attractive for possible future application as plant growth promoting inoculants for wheat production.

## Conclusion

Our findings suggest that the bacterial taxa involved in the provision of P in the rhizosphere of wheat plants depend on plant’s growth stage and only minor on farming system and climate under *in vitro* conditions. This observation clearly pleads for more studies at defined and yield-determining stages of plant growth under *in vitro* and *in vivo* conditions. We found that *Pseudomonas* species were dominant at the stem elongation stage, i.e., the peak of vegetative biomass production, while *Phyllobacterium* species dominated at the grain filling stage (generative stage). This is the first time that the two isolated *Phyllobacterium* species are reported for their high P solubilization potential. Drought tolerance potentials of the three dominant genera were found to be increased at the second growth stage studied in July compared to the first one in May indicating an adaptation to drought over time and a higher tolerance in summer months. *Phyllobacterium* species expressed highest drought tolerance potential among isolated dominant phosphate solubilizing rhizobacteria. Therefore, further work should concentrate on functionality and abundance of this genus in rhizospheres of plants. Especially in the context of climate change related summer drought, the observed trait combination of *Phyllobacterium* species in this study may be of particular importance to adapt agriculture to dryer conditions in the future.

## Data Availability Statement

The raw data supporting the conclusions of this manuscript will be made available by the authors, without undue reservation, to any qualified researcher.

## Author Contributions

All authors conceived and designed the experiments, obtained funding, interpreted the results, and contributed to revisions and approved submission of the manuscript. TR and CB performed the field experiments. CB performed the laboratory works and data analysis. MT, TR, and CB wrote the manuscript with input from FB.

## Conflict of Interest

The authors declare that the research was conducted in the absence of any commercial or financial relationships that could be construed as a potential conflict of interest.
