# Supplementary material for: Shifts Between and Among Populations of Wheat Rhizosphere Pseudomonas, Streptomyces and Phyllobacterium Suggest Consistent Phosphate Mobilization at Different Wheat Growth Stages Under Abiotic Stress
Source: Front Microbiol. 2020 Jan 22;10:3109. doi: 10.3389/fmicb.2019.03109 (PMC6987145; doi:10.3389/fmicb.2019.03109)
Supplement: Supplementary file 1 [file Data_Sheet_1.docx]

Supplementary Material

# Supplementary Figures


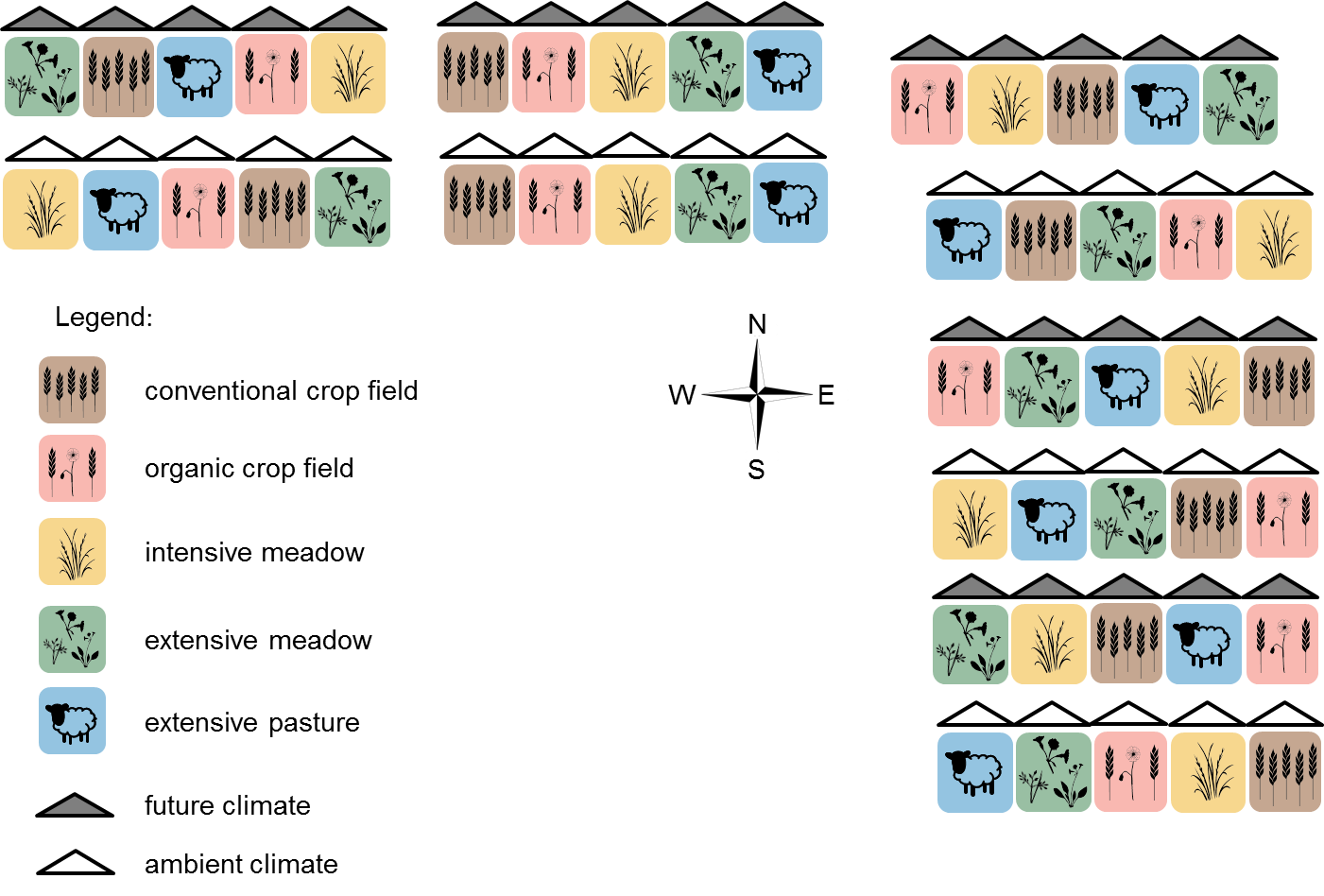


Supplementary Figure 1: Experimental design of the Global Change Experimental Facility (GCEF). The facility includes 50 plots (subplots) arranged in ten blocks (mainplots). Five blocks are attributed to ambient and future climate, respectively, whereby each block comprises all five land use types. Copyrights of land use icons are held by Gottschall/Siebert (Schädler et al. 2019).


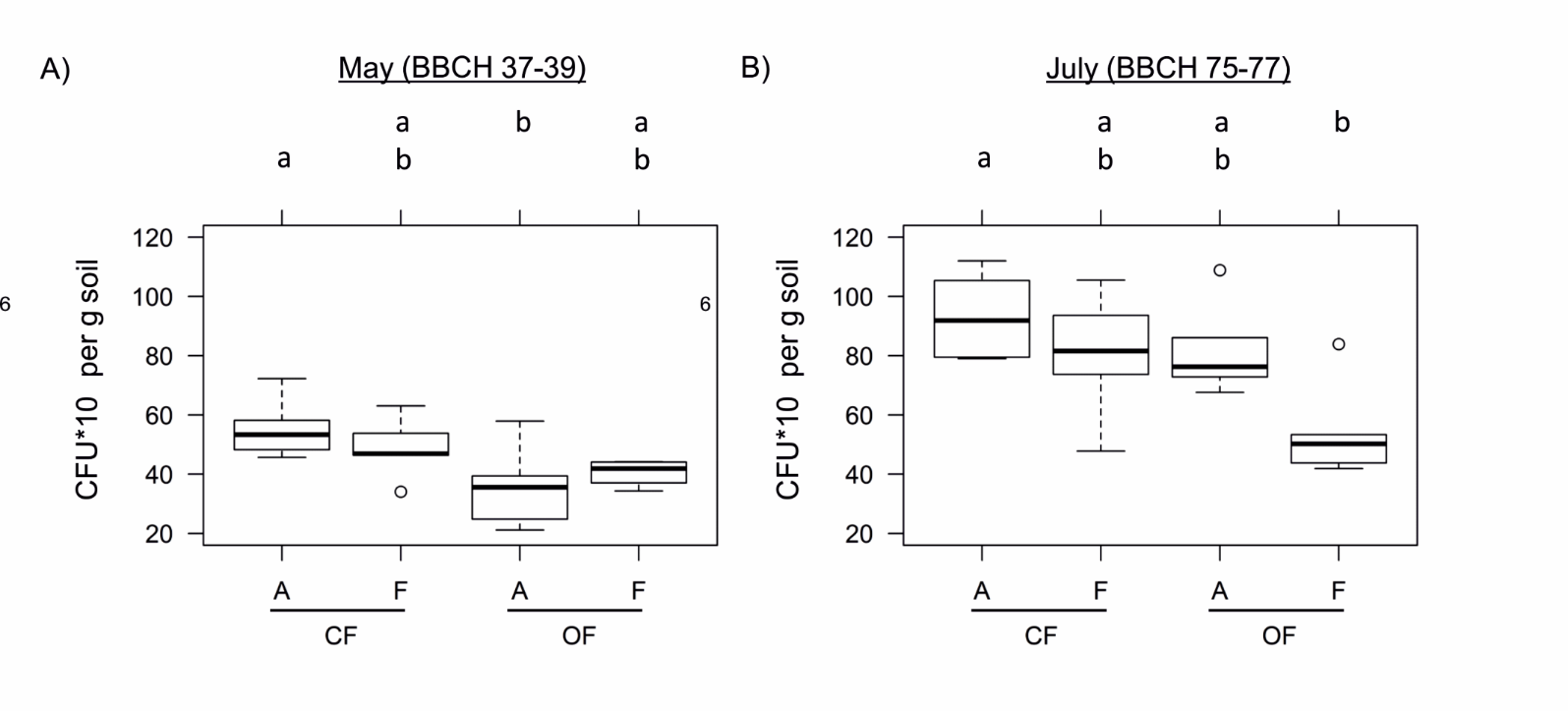


Supplementary Figure 2: Abundance of phosphate solubilizing rhizobacteria. Colony forming units of wheat rhizobacteria per g of soil, grown on Pikovskaya agar plates, in A) May 2015 sampling at BBCH stage 37-39 and in B) July 2015 sampling, at BBCH stage 75-77. Significant differences between treatments - ambient (A) and future (F) climate; conventional (CF) and organic (OF) farming system - for each time point are marked by different small letters.


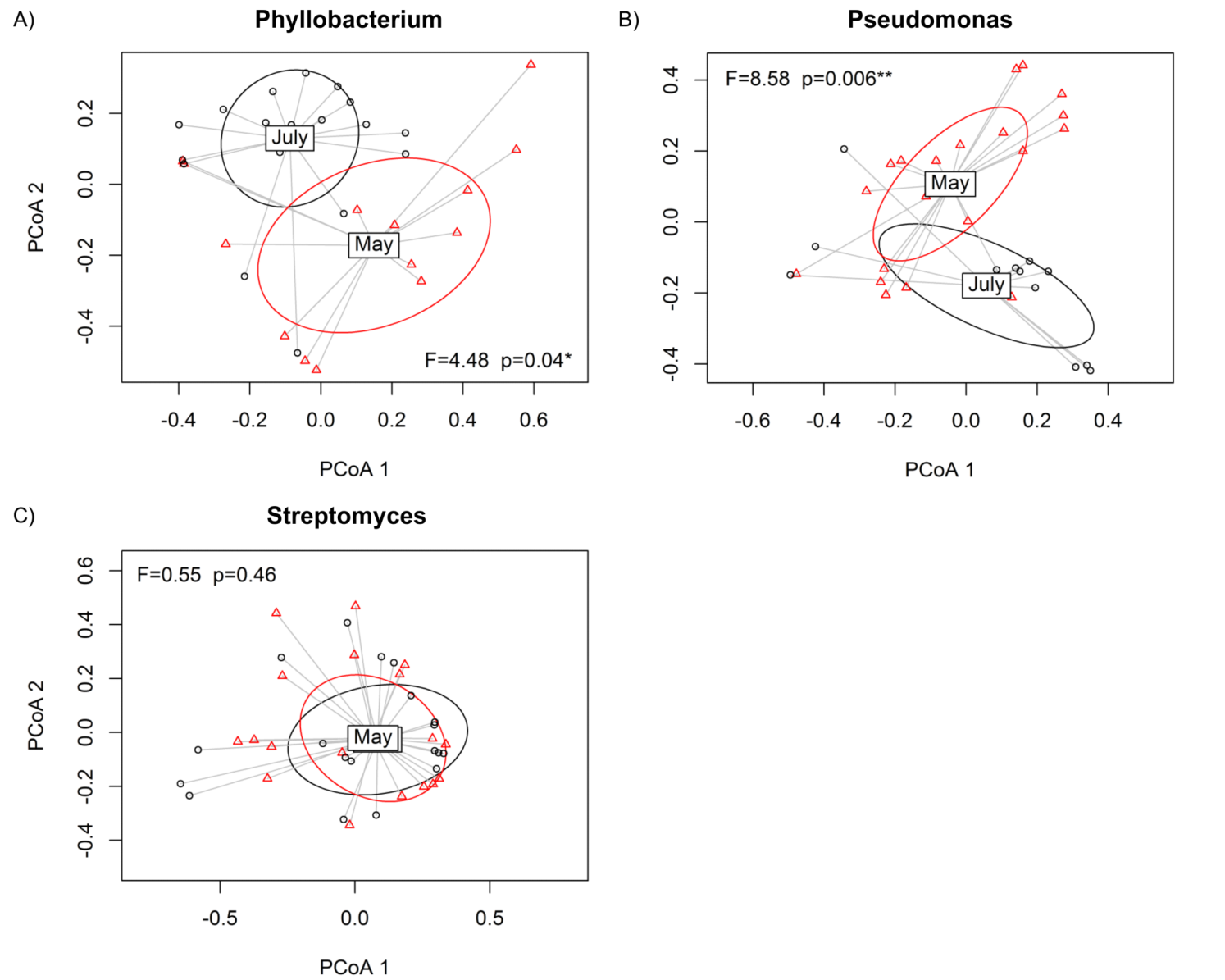


Supplementary Figure 3: Effect of wheat growth stage on A) *Phyllobacterium*, B) *Pseudomonas* and C) *Streptomyces* isolates structure. Dispersion patterns running betadisper function were calculated on distance matrix using method of Bray Curtis, followed by ANOVA testing for differences in composition of isolated species between the two sampling points, May (stem elongation, BBCH 37-39, red triangles) and July (grain filling, BBCH 75-77, black circles) respectively.


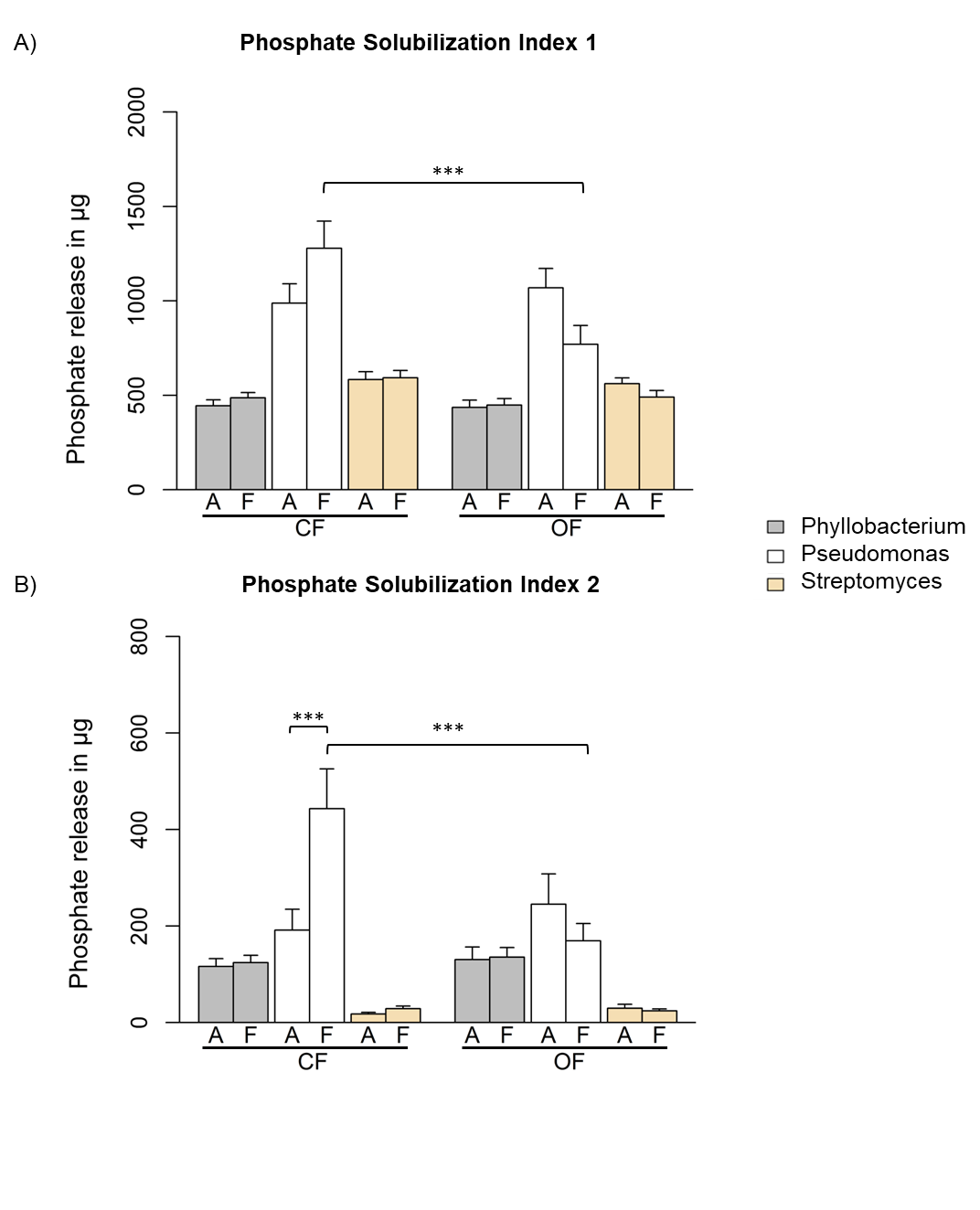


Supplementary Figure 4: Impact of climate and farming system on phosphate solubilization potentials of the three most dominant genera. Potentials are given for *Phyllobacterium*, *Pseudomonas* and *Streptomyces* calculated with A) PSI 1 and B) PSI 2, and isolated from conventional farming (CF) and organic farming (OF) under ambient (A) and future (F) climate conditions. Significant interactions within each genus are marked with p<0.001***.


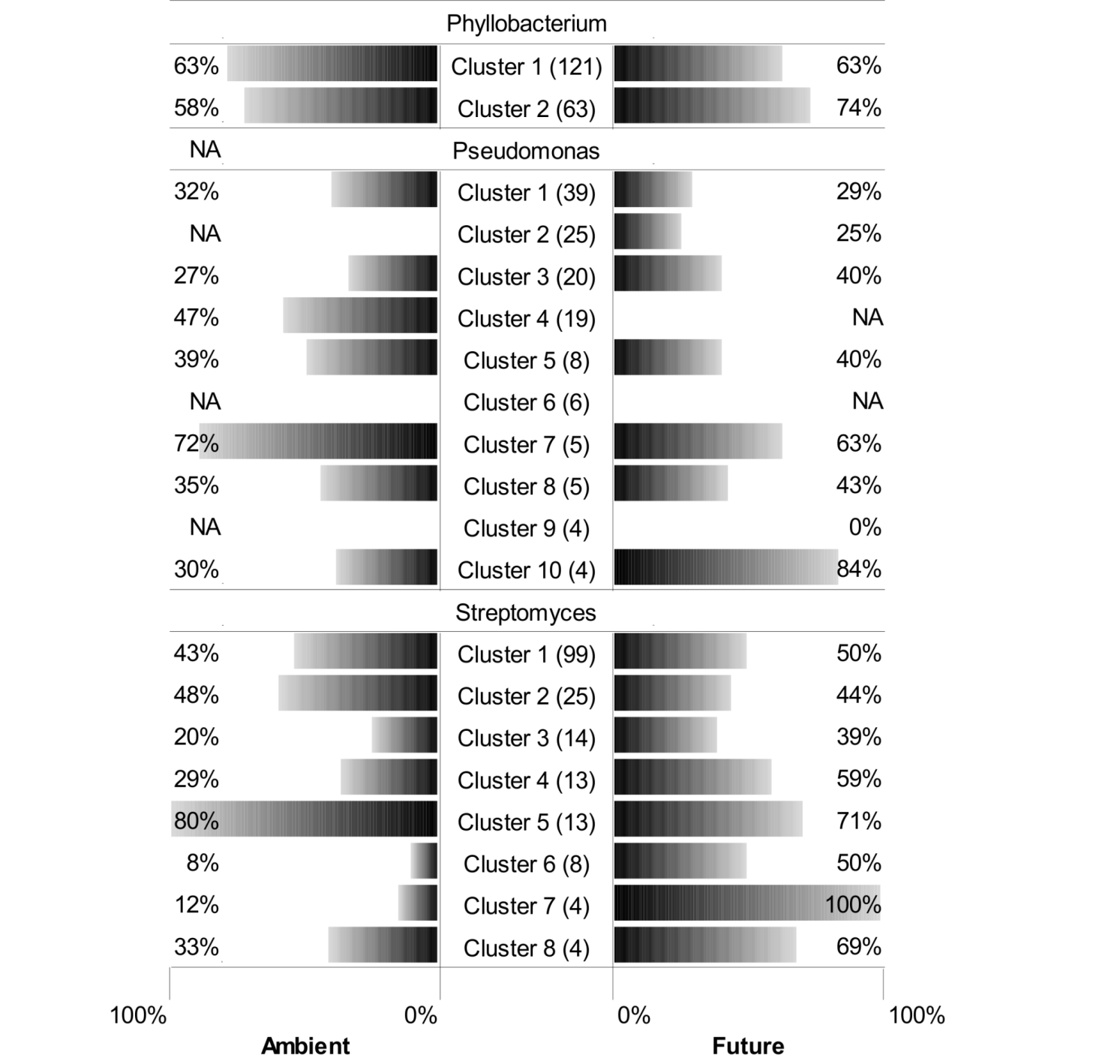


Supplementary Figure 5: Drought resistance under ambient and future climate conditions among the predominant phylogenetic clusters of *Phyllobacterium*, *Pseudomonas* and *Streptomyces*. Number of isolates for each cluster is given in parenthesis. Respective clusters marked with NA contain isolates that did not grow, neither on control (YME) nor on test media (YME+PEG).

# Supplementary Tables

Supplementary Table 1: Total number, origin and description of isolates cultivated on Pikovskaya medium. Isolates were gained at two different sampling points (May and July 2015), from two different farming systems (CF-conventional farming, OF-organic farming) and climate treatments (A-ambient climate, F-future climate). Number of base pairs (bp), assignment to genus and NCBI (National Center for Biotechnology Information-gene data bank) number is given.

| Isolates | Sampling | Genus | Size (bp) | Land use | Climate | Acc. Number (NCBI) |
| --- | --- | --- | --- | --- | --- | --- |
| PN2-B01P2-1 | May 2015 | *Pseudomonas sp.* | 1086 | CF | A | MK637864 |
| PN2-B01P2-2 | May 2015 | *Pseudomonas sp.* | 1057 | CF | A | MK637859 |
| PN2-B01P2-3 | May 2015 | *Pseudomonas sp.* | 1057 | CF | A | MK637861 |
| PN2-B01P2-4 | May 2015 | *Pseudomonas sp.* | 1050 | CF | A | MK637884 |
| PN2-B01P2-5 | May 2015 | *Streptomyces sp.* | 1053 | CF | A | MK638553 |
| PN2-B01P2-6 | May 2015 | *Pseudomonas sp.* | 1045 | CF | A | MK637871 |
| PN2-B01P2-7 | May 2015 | *Variovorax sp.* | 1051 | CF | A | MK638652 |
| PN2-B01P2-8 | May 2015 | *Agrobacterium sp.* | 1078 | CF | A | MK638040 |
| PN2-B01P2-9 | May 2015 | *Pseudomonas sp.* | 1057 | CF | A | MK637870 |
| PN2-B01P2-10 | May 2015 | *Pseudomonas sp.* | 1057 | CF | A | MK637882 |
| PN2-B01P2-11 | May 2015 | *Pseudomonas sp.* | 1057 | CF | A | MK637867 |
| PN2-B01P2-12 | May 2015 | *Pseudomonas sp.* | 1057 | CF | A | MK637869 |
| PN2-B01P2-13 | May 2015 | *Dyella sp.* | 1063 | CF | A | MK638116 |
| PN2-B01P2-14 | May 2015 | *Dyella sp.* | 1059 | CF | A | MK638115 |
| PN2-B01P2-15 | May 2015 | *Mesorhizobium sp.* | 1011 | CF | A | MK638184 |
| PN2-B01P2-16 | May 2015 | *Pseudomonas sp.* | 1057 | CF | A | MK637865 |
| PN2-B01P2-17 | May 2015 | *Rhizobium sp.* | 1036 | CF | A | MK638386 |
| PN2-B01P2-18 | May 2015 | *Mesorhizobium sp.* | 1037 | CF | A | MK638154 |
| PN2-B01P2-19 | May 2015 | *Streptomyces sp.* | 1023 | CF | A | MK638528 |
| PN2-B01P2-20 | May 2015 | *Mesorhizobium sp.* | 1036 | CF | A | MK638159 |
| PN2-B01P4-1 | May 2015 | *Pseudomonas sp.* | 1057 | OF | A | MK637877 |
| PN2-B01P4-2 | May 2015 | *Massilia sp.* | 1062 | OF | A | MK638153 |
| PN2-B01P4-3 | May 2015 | *Pseudomonas sp.* | 1056 | OF | A | MK637855 |
| PN2-B01P4-4 | May 2015 | *Rhizobium sp.* | 1033 | OF | A | MK638383 |
| PN2-B01P4-5 | May 2015 | *Pseudomonas sp.* | 1057 | OF | A | MK637858 |
| PN2-B01P4-6 | May 2015 | *Streptomyces sp.* | 1018 | OF | A | MK638619 |
| PN2-B01P4-7 | May 2015 | *Streptomyces sp.* | 1039 | OF | A | MK638454 |
| PN2-B01P4-8 | May 2015 | *Streptomyces sp.* | 1019 | OF | A | MK638482 |
| PN2-B01P4-9 | May 2015 | *Streptomyces sp.* | 1086 | OF | A | MK638574 |
| PN2-B01P4-10 | May 2015 | *Streptomyces sp.* | 997 | OF | A | MK638597 |
| PN2-B01P4-12 | May 2015 | *Streptomyces sp.* | 1018 | OF | A | MK638618 |
| PN2-B01P4-13 | May 2015 | *Mesorhizobium sp.* | 1037 | OF | A | MK638157 |
| PN2-B01P4-14 | May 2015 | *Streptomyces sp.* | 1049 | OF | A | MK638545 |
| PN2-B01P4-15 | May 2015 | *Mesorhizobium sp.* | 1037 | OF | A | MK638158 |
| PN2-B01P4-16 | May 2015 | *Burkholderia sp.* | 1043 | OF | A | MK638086 |
| PN2-B01P4-17 | May 2015 | *Streptomyces sp.* | 924 | OF | A | MK638646 |
| PN2-B01P4-18 | May 2015 | *Bradyrhizobium sp.* | 600 | OF | A | MK638085 |
| PN2-B01P4-19 | May 2015 | *Mesorhizobium sp.* | 662 | OF | A | MK638185 |
| PN2-B01P4-20 | May 2015 | *Phyllobacterium sp.* | 746 | OF | A | MK638374 |
| PN2-B01P4-21 | May 2015 | *Rhizobium sp.* | 1033 | OF | A | MK638385 |
| PN2-B01P4-22 | May 2015 | *Streptomyces sp.* | 1080 | OF | A | MK638468 |
| PN2-B01P4-24 | May 2015 | *Phyllobacterium sp.* | 1037 | OF | A | MK638203 |
| PN2-B02P3-1 | May 2015 | *Pseudomonas sp.* | 1061 | OF | F | MK637881 |
| PN2-B02P3-2 | May 2015 | *Stenotrophomonas sp.* | 1034 | OF | F | MK638444 |
| PN2-B02P3-3 | May 2015 | *Pseudomonas sp.* | 1049 | OF | F | MK637868 |
| PN2-B02P3-4 | May 2015 | *Pseudomonas sp.* | 1060 | OF | F | MK637872 |
| PN2-B02P3-5 | May 2015 | *Pseudomonas sp.* | 1053 | OF | F | MK637862 |
| PN2-B02P3-6 | May 2015 | *Streptomyces sp.* | 1085 | OF | F | MK638470 |
| PN2-B02P3-7 | May 2015 | *Pseudomonas sp.* | 1057 | OF | F | MK637874 |
| PN2-B02P3-8 | May 2015 | *Streptomyces sp.* | 1049 | OF | F | MK638476 |
| PN2-B02P3-9 | May 2015 | *Arthrobacter sp.* | 1044 | OF | F | MK638053 |
| PN2-B02P3-10 | May 2015 | *Pseudomonas sp.* | 1057 | OF | F | MK637879 |
| PN2-B02P3-11 | May 2015 | *Tardiphaga sp.* | 1068 | OF | F | MK638648 |
| PN2-B02P3-12 | May 2015 | *Stenotrophomonas sp.* | 1087 | OF | F | MK638445 |
| PN2-B02P3-13 | May 2015 | *Pseudomonas sp.* | 1069 | OF | F | MK637856 |
| PN2-B02P3-14 | May 2015 | *Rhizobium sp.* | 1077 | OF | F | MK638384 |
| PN2-B02P3-15 | May 2015 | *Mesorhizobium sp.* | 1037 | OF | F | MK638156 |
| PN2-B02P3-16 | May 2015 | *Pseudomonas sp.* | 1057 | OF | F | MK637873 |
| PN2-B02P3-17 | May 2015 | *Streptomyces sp.* | 1042 | OF | F | MK638575 |
| PN2-B02P3-18 | May 2015 | *Dyadobacter sp.* | 1015 | OF | F | MK638112 |
| PN2-B02P3-19 | May 2015 | *Streptomyces sp.* | 1049 | OF | F | MK638452 |
| PN2-B02P3-20 | May 2015 | *Phyllobacterium sp.* | 1037 | OF | F | MK638202 |
| PN2-B02P3-22 | May 2015 | *Mesorhizobium sp.* | 1037 | OF | F | MK638155 |
| PN2-B02P4-1 | May 2015 | *Pseudomonas sp.* | 1069 | CF | F | MK637863 |
| PN2-B02P4-2 | May 2015 | *Pseudomonas sp.* | 1057 | CF | F | MK637875 |
| PN2-B02P4-3 | May 2015 | *Pseudomonas sp.* | 1057 | CF | F | MK637880 |
| PN2-B02P4-4 | May 2015 | *Buttiauxella sp.* | 1060 | CF | F | MK638100 |
| PN2-B02P4-5 | May 2015 | *Buttiauxella sp.* | 1060 | CF | F | MK638101 |
| PN2-B02P4-6 | May 2015 | *Pseudomonas sp.* | 1077 | CF | F | MK637853 |
| PN2-B02P4-7 | May 2015 | *Buttiauxella sp.* | 614 | CF | F | MK638102 |
| PN2-B02P4-8 | May 2015 | *Pseudomonas sp.* | 1057 | CF | F | MK637876 |
| PN2-B02P4-9 | May 2015 | *Pseudomonas sp.* | 1078 | CF | F | MK637854 |
| PN2-B02P4-10 | May 2015 | *Pseudomonas sp.* | 1057 | CF | F | MK637860 |
| PN2-B02P4-11 | May 2015 | *Pseudomonas sp.* | 1063 | CF | F | MK637878 |
| PN2-B02P4-12 | May 2015 | *Buttiauxella sp.* | 1083 | CF | F | MK638096 |
| PN2-B02P4-13 | May 2015 | *Pseudomonas sp.* | 1060 | CF | F | MK638009 |
| PN2-B02P4-14 | May 2015 | *Buttiauxella sp.* | 1068 | CF | F | MK638097 |
| PN2-B02P4-15 | May 2015 | *Pantoea sp.* | 1080 | CF | F | MK638201 |
| PN2-B02P4-16 | May 2015 | *Buttiauxella sp.* | 1068 | CF | F | MK638098 |
| PN2-B02P4-17 | May 2015 | *Pseudomonas sp.* | 1057 | CF | F | MK637866 |
| PN2-B02P4-18 | May 2015 | *Pseudomonas sp.* | 1069 | CF | F | MK637857 |
| PN2-B02P4-19 | May 2015 | *Buttiauxella sp.* | 1073 | CF | F | MK638099 |
| PN2-B02P4-20 | May 2015 | *Buttiauxella sp.* | 1038 | CF | F | MK638103 |
| PN2-B03P1-1 | May 2015 | *Pseudomonas sp.* | 1057 | CF | A | MK637883 |
| PN2-B03P1-2 | May 2015 | *Sphingomonas sp.* | 1034 | CF | A | MK638443 |
| PN2-B03P1-3 | May 2015 | *Agrobacterium sp.* | 604 | CF | A | MK638050 |
| PN2-B03P1-4 | May 2015 | *Agrobacterium sp.* | 1032 | CF | A | MK638045 |
| PN2-B03P1-5 | May 2015 | *Bacillus sp.* | 1068 | CF | A | MK638061 |
| PN2-B03P1-6 | May 2015 | *Bacillus sp.* | 883 | CF | A | MK638063 |
| PN2-B03P1-7 | May 2015 | *Streptomyces sp.* | 1063 | CF | A | MK638469 |
| PN2-B03P1-10 | May 2015 | *Pedobacter sp.* | 1013 | CF | A | MK638129 |
| PN2-B03P1-11 | May 2015 | *Rugamonas sp.* | 1059 | CF | A | MK638095 |
| PN2-B03P1-12 | May 2015 | *Streptomyces sp.* | 1080 | CF | A | MK638453 |
| PN2-B03P1-13 | May 2015 | *Rhodococcus sp.* | 1037 | CF | A | MK638438 |
| PN2-B03P1-14 | May 2015 | *Mesorhizobium sp.* | 1077 | CF | A | MK638160 |
| PN2-B03P1-15 | May 2015 | *Phyllobacterium sp.* | 841 | CF | A | MK638207 |
| PN2-B03P1-16 | May 2015 | *Caulobacter sp.* | 1069 | CF | A | MK638104 |
| PN2-B03P1-17 | May 2015 | *Phyllobacterium sp.* | 1070 | CF | A | MK638208 |
| PN2-B03P1-18 | May 2015 | *Streptomyces sp.* | 1072 | CF | A | MK638530 |
| PN2-B03P1-19 | May 2015 | *Streptomyces sp.* | 1087 | CF | A | MK638596 |
| PN2-B03P1-20 | May 2015 | *Rhizobium sp.* | 1076 | CF | A | MK638389 |
| PN2-B03P1-21 | May 2015 | *Rhizobium sp.* | 1066 | CF | A | MK638391 |
| PN2-B03P1-22 | May 2015 | *Streptomyces sp.* | 1018 | CF | A | MK638620 |
| PN2-B03P1-23 | May 2015 | *Agrobacterium sp.* | 1054 | CF | A | MK638046 |
| PN2-B03P1-24 | May 2015 | *Rhizobium sp.* | 1066 | CF | A | MK638394 |
| PN2-B03P1-25 | May 2015 | *Streptomyces sp.* | 1066 | CF | A | MK638529 |
| PN2-B03P2-1 | May 2015 | *Pseudomonas sp.* | 1072 | OF | A | MK637910 |
| PN2-B03P2-2 | May 2015 | *Pseudomonas sp.* | 1045 | OF | A | MK637908 |
| PN2-B03P2-3 | May 2015 | *Variovorax sp.* | 1079 | OF | A | MK638653 |
| PN2-B03P2-4 | May 2015 | *Arthrobacter sp.* | 1071 | OF | A | MK638057 |
| PN2-B03P2-5 | May 2015 | *Pseudomonas sp.* | 1056 | OF | A | MK637897 |
| PN2-B03P2-6 | May 2015 | *Arthrobacter sp.* | 1060 | OF | A | MK638054 |
| PN2-B03P2-7 | May 2015 | *Arthrobacter sp.* | 1071 | OF | A | MK638058 |
| PN2-B03P2-8 | May 2015 | *Pseudomonas sp.* | 1057 | OF | A | MK637902 |
| PN2-B03P2-9 | May 2015 | *Bacillus sp.* | 1068 | OF | A | MK638067 |
| PN2-B03P2-10 | May 2015 | *Bacillus sp.* | 1082 | OF | A | MK638066 |
| PN2-B03P2-11 | May 2015 | *Streptomyces sp.* | 1084 | OF | A | MK638539 |
| PN2-B03P2-12 | May 2015 | *Pseudomonas sp.* | 1069 | OF | A | MK637904 |
| PN2-B03P2-13 | May 2015 | *Streptomyces sp.* | 1090 | OF | A | MK638570 |
| PN2-B03P2-14 | May 2015 | *Streptomyces sp.* | 1090 | OF | A | MK638611 |
| PN2-B03P2-15 | May 2015 | *Streptomyces sp.* | 853 | OF | A | MK638531 |
| PN2-B03P2-16 | May 2015 | *Mesorhizobium sp.* | 1070 | OF | A | MK638162 |
| PN2-B03P2-17 | May 2015 | *Streptomyces sp.* | 1066 | OF | A | MK638475 |
| PN2-B03P2-18 | May 2015 | *Inquilinus sp.* | 1043 | OF | A | MK638134 |
| PN2-B03P2-19 | May 2015 | *Mesorhizobium sp.* | 596 | OF | A | MK638182 |
| PN2-B03P2-20 | May 2015 | *Streptomyces sp.* | 1066 | OF | A | MK638474 |
| PN2-B03P2-21 | May 2015 | *Mesorhizobium sp.* | 1083 | OF | A | MK638161 |
| PN2-B03P2-22 | May 2015 | *Streptomyces sp.* | 1084 | OF | A | MK638536 |
| PN2-B03P2-23 | May 2015 | *Mesorhizobium sp.* | 1070 | OF | A | MK638163 |
| PN2-B04P1-1 | May 2015 | *Pseudomonas sp.* | 1072 | CF | F | MK637891 |
| PN2-B04P1-2 | May 2015 | *Mucilaginibacter sp.* | 1083 | CF | F | MK638193 |
| PN2-B04P1-3 | May 2015 | *Streptomyces sp.* | 1072 | CF | F | MK638557 |
| PN2-B04P1-4 | May 2015 | *Streptomyces sp.* | 1085 | CF | F | MK638472 |
| PN2-B04P1-5 | May 2015 | *Rugamonas sp.* | 1061 | CF | F | MK638090 |
| PN2-B04P1-6 | May 2015 | *Chitinophaga sp.* | 1087 | CF | F | MK638106 |
| PN2-B04P1-7 | May 2015 | *Rhizobium sp.* | 1079 | CF | F | MK638388 |
| PN2-B04P1-8 | May 2015 | *Arthrobacter sp.* | 1057 | CF | F | MK638055 |
| PN2-B04P1-9 | May 2015 | *Chitinophaga sp.* | 1088 | CF | F | MK638107 |
| PN2-B04P1-10 | May 2015 | *Arthrobacter sp.* | 1084 | CF | F | MK638060 |
| PN2-B04P1-11 | May 2015 | *Streptomyces sp.* | 1018 | CF | F | MK638621 |
| PN2-B04P1-12 | May 2015 | *Rhizobium sp.* | 1061 | CF | F | MK638387 |
| PN2-B04P1-13 | May 2015 | *Phyllobacterium sp.* | 1079 | CF | F | MK638205 |
| PN2-B04P1-14 | May 2015 | *Streptomyces sp.* | 1090 | CF | F | MK638555 |
| PN2-B04P1-15 | May 2015 | *Bradyrhizobium sp.* | 1071 | CF | F | MK638082 |
| PN2-B04P1-16 | May 2015 | *Rhizobium sp.* | 1066 | CF | F | MK638393 |
| PN2-B04P1-17 | May 2015 | *Phyllobacterium sp.* | 1043 | CF | F | MK638206 |
| PN2-B04P1-18 | May 2015 | *Streptomyces sp.* | 1055 | CF | F | MK638569 |
| PN2-B04P1-19 | May 2015 | *Chryseobacterium sp.* | 1049 | CF | F | MK638108 |
| PN2-B04P1-20 | May 2015 | *Rhizobium sp.* | 1073 | CF | F | MK638392 |
| PN2-B04P2-1 | May 2015 | *Pseudomonas sp.* | 1072 | OF | F | MK637894 |
| PN2-B04P2-2 | May 2015 | *Pseudomonas sp.* | 1085 | OF | F | MK637888 |
| PN2-B04P2-3 | May 2015 | *Bacillus sp.* | 1076 | OF | F | MK638065 |
| PN2-B04P2-4 | May 2015 | *Burkholderia sp.* | 1051 | OF | F | MK638094 |
| PN2-B04P2-5 | May 2015 | *Bacillus sp.* | 1089 | OF | F | MK638064 |
| PN2-B04P2-6 | May 2015 | *Streptomyces sp.* | 1018 | OF | F | MK638622 |
| PN2-B04P2-7 | May 2015 | *Pseudomonas sp.* | 1066 | OF | F | MK637901 |
| PN2-B04P2-8 | May 2015 | *Streptomyces sp.* | 1018 | OF | F | MK638623 |
| PN2-B04P2-9 | May 2015 | *Streptomyces sp.* | 962 | OF | F | MK638537 |
| PN2-B04P2-10 | May 2015 | *Streptomyces sp.* | 1055 | OF | F | MK638573 |
| PN2-B04P2-11 | May 2015 | *Pseudomonas sp.* | 1083 | OF | F | MK637893 |
| PN2-B04P2-12 | May 2015 | *Pseudomonas sp.* | 1057 | OF | F | MK637892 |
| PN2-B04P2-13 | May 2015 | *Arthrobacter sp.* | 1078 | OF | F | MK638059 |
| PN2-B04P2-14 | May 2015 | *Mesorhizobium sp.* | 1054 | OF | F | MK638164 |
| PN2-B04P2-15 | May 2015 | *Streptomyces sp.* | 1055 | OF | F | MK638571 |
| PN2-B04P2-16 | May 2015 | *Streptomyces sp.* | 1055 | OF | F | MK638572 |
| PN2-B04P2-17 | May 2015 | *Ochrobactrum sp.* | 1062 | OF | F | MK638197 |
| PN2-B04P2-18 | May 2015 | *Streptomyces sp.* | 1076 | OF | F | MK638471 |
| PN2-B04P2-19 | May 2015 | *Phyllobacterium sp.* | 1078 | OF | F | MK638204 |
| PN2-B04P2-20 | May 2015 | *Rhizobium sp.* | 1074 | OF | F | MK638390 |
| PN2-B04P2-21 | May 2015 | *Paraburkholderia sp.* | 1061 | OF | F | MK638087 |
| PN2-B04P2-22 | May 2015 | *Ochrobactrum sp.* | 1053 | OF | F | MK638198 |
| PN2-B05P1-1 | May 2015 | *Pseudomonas sp.* | 1057 | OF | A | MK637896 |
| PN2-B05P1-2 | May 2015 | *Pseudomonas sp.* | 1056 | OF | A | MK637900 |
| PN2-B05P1-3 | May 2015 | *Pseudomonas sp.* | 1086 | OF | A | MK637889 |
| PN2-B05P1-4 | May 2015 | *Pseudomonas sp.* | 1085 | OF | A | MK637886 |
| PN2-B05P1-5 | May 2015 | *Pseudomonas sp.* | 1086 | OF | A | MK637887 |
| PN2-B05P1-6 | May 2015 | *Pseudomonas sp.* | 1057 | OF | A | MK637905 |
| PN2-B05P1-7 | May 2015 | *Pseudomonas sp.* | 1064 | OF | A | MK637906 |
| PN2-B05P1-8 | May 2015 | *Pseudomonas sp.* | 1069 | OF | A | MK637911 |
| PN2-B05P1-9 | May 2015 | *Pseudomonas sp.* | 1069 | OF | A | MK637907 |
| PN2-B05P1-10 | May 2015 | *Pseudomonas sp.* | 1075 | OF | A | MK637898 |
| PN2-B05P1-11 | May 2015 | *Pseudomonas sp.* | 1055 | OF | A | MK637890 |
| PN2-B05P1-12 | May 2015 | *Pseudomonas sp.* | 1071 | OF | A | MK637885 |
| PN2-B05P1-13 | May 2015 | *Pseudomonas sp.* | 1073 | OF | A | MK637899 |
| PN2-B05P1-14 | May 2015 | *Pseudomonas sp.* | 1086 | OF | A | MK637903 |
| PN2-B05P1-15 | May 2015 | *Pseudomonas sp.* | 1072 | OF | A | MK637895 |
| PN2-B05P1-16 | May 2015 | *Pseudomonas sp.* | 1066 | OF | A | MK637909 |
| PN2-B05P1-17 | May 2015 | *Streptomyces sp.* | 1090 | OF | A | MK638556 |
| PN2-B05P1-18 | May 2015 | *Plantibacter sp.* | 1084 | OF | A | MK638377 |
| PN2-B05P1-20 | May 2015 | *Rhizobium sp.* | 1066 | OF | A | MK638395 |
| PN2-B05P3-1 | May 2015 | *Pseudomonas sp.* | 1085 | CF | A | MK637917 |
| PN2-B05P3-2 | May 2015 | *Pseudomonas sp.* | 1086 | CF | A | MK637924 |
| PN2-B05P3-3 | May 2015 | *Pseudomonas sp.* | 1076 | CF | A | MK637952 |
| PN2-B05P3-4 | May 2015 | *Pseudomonas sp.* | 1085 | CF | A | MK637937 |
| PN2-B05P3-5 | May 2015 | *Pseudomonas sp.* | 1072 | CF | A | MK637945 |
| PN2-B05P3-6 | May 2015 | *Pseudomonas sp.* | 1081 | CF | A | MK637923 |
| PN2-B05P3-7 | May 2015 | *Pseudomonas sp.* | 1085 | CF | A | MK637935 |
| PN2-B05P3-8 | May 2015 | *Pseudomonas sp.* | 1077 | CF | A | MK637936 |
| PN2-B05P3-9 | May 2015 | *Pseudomonas sp.* | 1085 | CF | A | MK637944 |
| PN2-B05P3-10 | May 2015 | *Agrobacterium sp.* | 1068 | CF | A | MK638047 |
| PN2-B05P3-11 | May 2015 | *Streptomyces sp.* | 1012 | CF | A | MK638463 |
| PN2-B05P3-12 | May 2015 | *Streptomyces sp.* | 1085 | CF | A | MK638458 |
| PN2-B05P3-13 | May 2015 | *Phyllobacterium sp.* | 1071 | CF | A | MK638211 |
| PN2-B05P3-14 | May 2015 | *Phyllobacterium sp.* | 1070 | CF | A | MK638213 |
| PN2-B05P3-15 | May 2015 | *Pseudomonas sp.* | 1076 | CF | A | MK637919 |
| PN2-B05P3-16 | May 2015 | *Rugamonas sp.* | 1047 | CF | A | MK638091 |
| PN2-B05P3-17 | May 2015 | *Achromobacter sp.* | 1017 | CF | A | MK638022 |
| PN2-B05P3-18 | May 2015 | *Phyllobacterium sp.* | 1068 | CF | A | MK638399 |
| PN2-B05P3-19 | May 2015 | *Streptomyces sp.* | 1087 | CF | A | MK638568 |
| PN2-B05P3-20 | May 2015 | *Phyllobacterium sp.* | 1021 | CF | A | MK638216 |
| PN2-B05P3-21 | May 2015 | *Agromyces sp.* | 1072 | CF | A | MK638052 |
| PN2-B06P2-1 | May 2015 | *Pseudomonas sp.* | 1076 | CF | F | MK637941 |
| PN2-B06P2-2 | May 2015 | *Pseudomonas sp.* | 1066 | CF | F | MK637939 |
| PN2-B06P2-3 | May 2015 | *Pseudomonas sp.* | 1072 | CF | F | MK637930 |
| PN2-B06P2-4 | May 2015 | *Pseudomonas sp.* | 1077 | CF | F | MK637942 |
| PN2-B06P2-5 | May 2015 | *Pseudomonas sp.* | 1053 | CF | F | MK637940 |
| PN2-B06P2-6 | May 2015 | *Pseudomonas sp.* | 1085 | CF | F | MK637926 |
| PN2-B06P2-7 | May 2015 | *Pseudomonas sp.* | 1085 | CF | F | MK637949 |
| PN2-B06P2-8 | May 2015 | *Bacillus sp.* | 280 | CF | F | MK638081 |
| PN2-B06P2-9 | May 2015 | *Pseudomonas sp.* | 1085 | CF | F | MK637914 |
| PN2-B06P2-10 | May 2015 | *Pseudomonas sp.* | 1082 | CF | F | MK637951 |
| PN2-B06P2-11 | May 2015 | *Pseudomonas sp.* | 1073 | CF | F | MK637913 |
| PN2-B06P2-12 | May 2015 | *Pseudomonas sp.* | 1081 | CF | F | MK637922 |
| PN2-B06P2-14 | May 2015 | *Pseudomonas sp.* | 1077 | CF | F | MK637928 |
| PN2-B06P2-15 | May 2015 | *Pseudomonas sp.* | 1085 | CF | F | MK637931 |
| PN2-B06P2-16 | May 2015 | *Phyllobacterium sp.* | 1070 | CF | F | MK638212 |
| PN2-B06P2-17 | May 2015 | *Streptomyces sp.* | 1086 | CF | F | MK638567 |
| PN2-B06P2-19 | May 2015 | *Pseudomonas sp.* | 1080 | CF | F | MK637920 |
| PN2-B06P2-20 | May 2015 | *Phyllobacterium sp.* | 1079 | CF | F | MK638210 |
| PN2-B06P4-1 | May 2015 | *Pseudomonas sp.* | 1072 | OF | F | MK637925 |
| PN2-B06P4-2 | May 2015 | *Pseudomonas sp.* | 1084 | OF | F | MK637933 |
| PN2-B06P4-3 | May 2015 | *Pseudomonas sp.* | 1077 | OF | F | MK637929 |
| PN2-B06P4-4 | May 2015 | *Pseudomonas sp.* | 1057 | OF | F | MK637934 |
| PN2-B06P4-5 | May 2015 | *Serratia sp.* | 1039 | OF | F | MK638441 |
| PN2-B06P4-6 | May 2015 | *Pseudomonas sp.* | 1072 | OF | F | MK637921 |
| PN2-B06P4-7 | May 2015 | *Streptomyces sp.* | 1070 | OF | F | MK638459 |
| PN2-B06P4-8 | May 2015 | *Streptomyces sp.* | 1080 | OF | F | MK638461 |
| PN2-B06P4-9 | May 2015 | *Bacillus sp.* | 1094 | OF | F | MK638068 |
| PN2-B06P4-10 | May 2015 | *Streptomyces sp.* | 1070 | OF | F | MK638595 |
| PN2-B06P4-11 | May 2015 | *Pseudomonas sp.* | 1072 | OF | F | MK637918 |
| PN2-B06P4-12 | May 2015 | *Streptomyces sp.* | 1085 | OF | F | MK638457 |
| PN2-B06P4-13 | May 2015 | *Pseudomonas sp.* | 1085 | OF | F | MK637915 |
| PN2-B06P4-14 | May 2015 | *Phyllobacterium sp.* | 1066 | OF | F | MK638396 |
| PN2-B06P4-16 | May 2015 | *Phyllobacterium sp.* | 1070 | OF | F | MK638214 |
| PN2-B06P4-17 | May 2015 | *Streptomyces sp.* | 1085 | OF | F | MK638473 |
| PN2-B06P4-18 | May 2015 | *Streptomyces sp.* | 1064 | OF | F | MK638593 |
| PN2-B06P4-19 | May 2015 | *Streptomyces sp.* | 1079 | OF | F | MK638612 |
| PN2-B06P4-20 | May 2015 | *Streptomyces sp.* | 1010 | OF | F | MK638462 |
| PN2-B06P4-22 | May 2015 | *Phyllobacterium sp.* | 1068 | OF | F | MK638397 |
| PN2-B07P1-1 | May 2015 | *Pseudomonas sp.* | 1067 | OF | F | MK637954 |
| PN2-B07P1-2 | May 2015 | *Pseudomonas sp.* | 1070 | OF | F | MK637953 |
| PN2-B07P1-3 | May 2015 | *Pseudomonas sp.* | 1076 | OF | F | MK637956 |
| PN2-B07P1-4 | May 2015 | *Pseudomonas sp.* | 1029 | OF | F | MK637946 |
| PN2-B07P1-5 | May 2015 | *Pseudomonas sp.* | 1070 | OF | F | MK637943 |
| PN2-B07P1-6 | May 2015 | *Pseudomonas sp.* | 1069 | OF | F | MK637947 |
| PN2-B07P1-7 | May 2015 | *Rugamonas sp.* | 1068 | OF | F | MK638092 |
| PN2-B07P1-8 | May 2015 | *Pseudomonas sp.* | 1073 | OF | F | MK637912 |
| PN2-B07P1-9 | May 2015 | *Rhizobium sp.* | 1025 | OF | F | MK638400 |
| PN2-B07P1-10 | May 2015 | *Rhodococcus sp.* | 1078 | OF | F | MK638436 |
| PN2-B07P1-11 | May 2015 | *Streptomyces sp.* | 1085 | OF | F | MK638565 |
| PN2-B07P1-12 | May 2015 | *Pseudomonas sp.* | 1072 | OF | F | MK637916 |
| PN2-B07P1-13 | May 2015 | *Streptomyces sp.* | 1085 | OF | F | MK638566 |
| PN2-B07P1-14 | May 2015 | *Pseudomonas sp.* | 466 | OF | F | MK638010 |
| PN2-B07P1-15 | May 2015 | *Ochrobactrum sp.* | 1049 | OF | F | MK638199 |
| PN2-B07P1-16 | May 2015 | *Pseudomonas sp.* | 1077 | OF | F | MK637927 |
| PN2-B07P1-17 | May 2015 | *Rhizobium sp.* | 1062 | OF | F | MK638401 |
| PN2-B07P1-18 | May 2015 | *Plantibacter sp.* | 1076 | OF | F | MK638378 |
| PN2-B07P1-19 | May 2015 | *Streptomyces sp.* | 1060 | OF | F | MK638460 |
| PN2-B07P1-20 | May 2015 | *Variovorax sp.* | 1031 | OF | F | MK638654 |
| PN2-B07P5-1 | May 2015 | *Tsukamurella sp.* | 1058 | CF | F | MK638650 |
| PN2-B07P5-3 | May 2015 | *Streptomyces sp.* | 1085 | CF | F | MK638456 |
| PN2-B07P5-4 | May 2015 | *Pseudomonas sp.* | 1067 | CF | F | MK637938 |
| PN2-B07P5-5 | May 2015 | *Pseudomonas sp.* | 1085 | CF | F | MK637950 |
| PN2-B07P5-6 | May 2015 | *Tsukamurella sp.* | 1076 | CF | F | MK638651 |
| PN2-B07P5-7 | May 2015 | *Pseudomonas sp.* | 1077 | CF | F | MK637932 |
| PN2-B07P5-8 | May 2015 | *Pseudomonas sp.* | 1078 | CF | F | MK637948 |
| PN2-B07P5-9 | May 2015 | *Streptomyces sp.* | 1085 | CF | F | MK638467 |
| PN2-B07P5-10 | May 2015 | *Dyadobacter sp.* | 1071 | CF | F | MK638114 |
| PN2-B07P5-11 | May 2015 | *Pseudomonas sp.* | 1076 | CF | F | MK637955 |
| PN2-B07P5-12 | May 2015 | *Dyadobacter sp.* | 1040 | CF | F | MK638113 |
| PN2-B07P5-13 | May 2015 | *Phyllobacterium sp.* | 1065 | CF | F | MK638215 |
| PN2-B07P5-14 | May 2015 | *Streptomyces sp.* | 1018 | CF | F | MK638624 |
| PN2-B07P5-15 | May 2015 | *Phyllobacterium sp.* | 1070 | CF | F | MK638219 |
| PN2-B07P5-16 | May 2015 | *Mesorhizobium sp.* | 1080 | CF | F | MK638165 |
| PN2-B07P5-17 | May 2015 | *Streptomyces sp.* | 673 | CF | F | MK638647 |
| PN2-B07P5-18 | May 2015 | *Phyllobacterium sp.* | 1077 | CF | F | MK638224 |
| PN2-B07P5-20 | May 2015 | *Phyllobacterium sp.* | 1066 | CF | F | MK638225 |
| PN2-B07P5-21 | May 2015 | *Streptomyces sp.* | 1076 | CF | F | MK638532 |
| PN2-B07P5-22 | May 2015 | *Phyllobacterium sp.* | 1070 | CF | F | MK638221 |
| PN2-B07P5-23 | May 2015 | *Phyllobacterium sp.* | 1070 | CF | F | MK638223 |
| PN2-B07P5-24 | May 2015 | *Phyllobacterium sp.* | 1077 | CF | F | MK638217 |
| PN2-B08P4-1 | May 2015 | *Pseudomonas sp.* | 1074 | CF | A | MK637962 |
| PN2-B08P4-2 | May 2015 | *Pseudomonas sp.* | 1072 | CF | A | MK637967 |
| PN2-B08P4-3 | May 2015 | *Pseudomonas sp.* | 1070 | CF | A | MK637976 |
| PN2-B08P4-4 | May 2015 | *Pseudomonas sp.* | 1076 | CF | A | MK637960 |
| PN2-B08P4-5 | May 2015 | *Pseudomonas sp.* | 1073 | CF | A | MK637963 |
| PN2-B08P4-6 | May 2015 | *Pseudomonas sp.* | 1077 | CF | A | MK637971 |
| PN2-B08P4-7 | May 2015 | *Pseudomonas sp.* | 1077 | CF | A | MK637973 |
| PN2-B08P4-8 | May 2015 | *Pseudomonas sp.* | 1072 | CF | A | MK637984 |
| PN2-B08P4-9 | May 2015 | *Pseudomonas sp.* | 766 | CF | A | MK637990 |
| PN2-B08P4-10 | May 2015 | *Pseudomonas sp.* | 1074 | CF | A | MK637977 |
| PN2-B08P4-11 | May 2015 | *Pseudomonas sp.* | 1072 | CF | A | MK637969 |
| PN2-B08P4-12 | May 2015 | *Pseudomonas sp.* | 1072 | CF | A | MK637983 |
| PN2-B08P4-13 | May 2015 | *Pseudomonas sp.* | 1077 | CF | A | MK637975 |
| PN2-B08P4-14 | May 2015 | *Pseudomonas sp.* | 1073 | CF | A | MK637961 |
| PN2-B08P4-15 | May 2015 | *Serratia sp.* | 1081 | CF | A | MK638442 |
| PN2-B08P4-16 | May 2015 | *Pseudomonas sp.* | 1073 | CF | A | MK637982 |
| PN2-B08P4-17 | May 2015 | *Pseudomonas sp.* | 1074 | CF | A | MK637957 |
| PN2-B08P4-18 | May 2015 | *Microbacterium sp.* | 303 | CF | A | MK638190 |
| PN2-B08P4-19 | May 2015 | *Pseudomonas sp.* | 1085 | CF | A | MK637972 |
| PN2-B08P4-20 | May 2015 | *Pseudomonas sp.* | 1077 | CF | A | MK637965 |
| PN2-B08P5-1 | May 2015 | *Pseudomonas sp.* | 1072 | OF | A | MK637986 |
| PN2-B08P5-2 | May 2015 | *Pseudomonas sp.* | 1086 | OF | A | MK637970 |
| PN2-B08P5-3 | May 2015 | *Pseudomonas sp.* | 1079 | OF | A | MK637966 |
| PN2-B08P5-4 | May 2015 | *Variovorax sp.* | 1067 | OF | A | MK638655 |
| PN2-B08P5-5 | May 2015 | *Pseudomonas sp.* | 1074 | OF | A | MK637974 |
| PN2-B08P5-6 | May 2015 | *Pseudomonas sp.* | 1072 | OF | A | MK637985 |
| PN2-B08P5-7 | May 2015 | *Pseudomonas sp.* | 1072 | OF | A | MK637980 |
| PN2-B08P5-8 | May 2015 | *Pseudomonas sp.* | 1072 | OF | A | MK637981 |
| PN2-B08P5-9 | May 2015 | *Rhodococcus sp.* | 721 | OF | A | MK638440 |
| PN2-B08P5-10 | May 2015 | *Pseudomonas sp.* | 1072 | OF | A | MK637979 |
| PN2-B08P5-11 | May 2015 | *Rhizobium sp.* | 1062 | OF | A | MK638404 |
| PN2-B08P5-12 | May 2015 | *Rhodococcus sp.* | 1030 | OF | A | MK638437 |
| PN2-B08P5-13 | May 2015 | *Flavobacterium sp.* | 1053 | OF | A | MK638130 |
| PN2-B08P5-14 | May 2015 | *Phyllobacterium sp.* | 1070 | OF | A | MK638232 |
| PN2-B08P5-15 | May 2015 | *Streptomyces sp.* | 1073 | OF | A | MK638466 |
| PN2-B08P5-16 | May 2015 | *Ensifer sp.* | 1070 | OF | A | MK638118 |
| PN2-B08P5-17 | May 2015 | *Phyllobacterium sp.* | 1070 | OF | A | MK638218 |
| PN2-B08P5-18 | May 2015 | *Phyllobacterium sp.* | 1066 | OF | A | MK638402 |
| PN2-B08P5-19 | May 2015 | *Rhizobium sp.* | 1066 | OF | A | MK638403 |
| PN2-B08P5-20 | May 2015 | *Streptomyces sp.* | 1056 | OF | A | MK638576 |
| PN2-B08P5-21 | May 2015 | *Phyllobacterium sp.* | 1070 | OF | A | MK638230 |
| PN2-B09P3-1 | May 2015 | *Streptomyces sp.* | 1076 | CF | F | MK638479 |
| PN2-B09P3-2 | May 2015 | *Pseudomonas sp.* | 1072 | CF | F | MK637964 |
| PN2-B09P3-3 | May 2015 | *Streptomyces sp.* | 1066 | CF | F | MK638522 |
| PN2-B09P3-4 | May 2015 | *Rugamonas sp.* | 1071 | CF | F | MK638093 |
| PN2-B09P3-5 | May 2015 | *Streptomyces sp.* | 1079 | CF | F | MK638465 |
| PN2-B09P3-7 | May 2015 | *Arthrobacter sp.* | 1093 | CF | F | MK638056 |
| PN2-B09P3-8 | May 2015 | *Streptomyces sp.* | 1070 | CF | F | MK638480 |
| PN2-B09P3-9 | May 2015 | *Rhodococcus sp.* | 1069 | CF | F | MK638439 |
| PN2-B09P3-10 | May 2015 | *Streptomyces sp.* | 1056 | CF | F | MK638592 |
| PN2-B09P3-11 | May 2015 | *Streptomyces sp.* | 1073 | CF | F | MK638598 |
| PN2-B09P3-13 | May 2015 | *Flavobacterium sp.* | 1040 | CF | F | MK638131 |
| PN2-B09P3-14 | May 2015 | *Phyllobacterium sp.* | 1070 | CF | F | MK638227 |
| PN2-B09P3-15 | May 2015 | *Streptomyces sp.* | 1070 | CF | F | MK638521 |
| PN2-B09P3-16 | May 2015 | *Phyllobacterium sp.* | 1068 | CF | F | MK638228 |
| PN2-B09P3-17 | May 2015 | *Phyllobacterium sp.* | 1068 | CF | F | MK638229 |
| PN2-B09P3-18 | May 2015 | *Phyllobacterium sp.* | 1068 | CF | F | MK638226 |
| PN2-B09P3-19 | May 2015 | *Microbacterium sp.* | 1077 | CF | F | MK638186 |
| PN2-B09P3-21 | May 2015 | *Phyllobacterium sp.* | 1065 | CF | F | MK638231 |
| PN2-B09P3-22 | May 2015 | *Streptomyces sp.* | 1076 | CF | F | MK638481 |
| PN2-B09P3-24 | May 2015 | *Phyllobacterium sp.* | 1070 | CF | F | MK638222 |
| PN2-B09P5-1 | May 2015 | *Pseudomonas sp.* | 1073 | OF | F | MK637978 |
| PN2-B09P5-2 | May 2015 | *Plantibacter sp.* | 1071 | OF | F | MK638380 |
| PN2-B09P5-3 | May 2015 | *Pseudomonas sp.* | 1074 | OF | F | MK637968 |
| PN2-B09P5-4 | May 2015 | *Pseudomonas sp.* | 1074 | OF | F | MK637958 |
| PN2-B09P5-5 | May 2015 | *Agrobacterium sp.* | 1073 | OF | F | MK638044 |
| PN2-B09P5-6 | May 2015 | *Streptomyces sp.* | 1087 | OF | F | MK638464 |
| PN2-B09P5-7 | May 2015 | *Bacillus sp.* | 1073 | OF | F | MK638071 |
| PN2-B09P5-8 | May 2015 | *Pseudomonas sp.* | 1049 | OF | F | MK637989 |
| PN2-B09P5-9 | May 2015 | *Flavobacterium sp.* | 907 | OF | F | MK638132 |
| PN2-B09P5-10 | May 2015 | *Pseudomonas sp.* | 1045 | OF | F | MK637988 |
| PN2-B09P5-11 | May 2015 | *Bacillus sp.* | 1072 | OF | F | MK638070 |
| PN2-B09P5-12 | May 2015 | *Agrobacterium sp.* | 1029 | OF | F | MK638043 |
| PN2-B09P5-13 | May 2015 | *Pseudomonas sp.* | 806 | OF | F | MK637987 |
| PN2-B09P5-14 | May 2015 | *Streptomyces sp.* | 1085 | OF | F | MK638519 |
| PN2-B09P5-15 | May 2015 | *Phyllobacterium sp.* | 1066 | OF | F | MK638220 |
| PN2-B09P5-16 | May 2015 | *Streptomyces sp.* | 1066 | OF | F | MK638477 |
| PN2-B09P5-17 | May 2015 | *Plantibacter sp.* | 1057 | OF | F | MK638379 |
| PN2-B09P5-18 | May 2015 | *Bacillus sp.* | 1076 | OF | F | MK638069 |
| PN2-B09P5-21 | May 2015 | *Plantibacter sp.* | 1015 | OF | F | MK638381 |
| PN2-B09P5-22 | May 2015 | *Phyllobacterium sp.* | 1026 | OF | F | MK638359 |
| PN2-B10P3-1 | May 2015 | *Pseudomonas sp.* | 1043 | OF | A | MK638012 |
| PN2-B10P3-2 | May 2015 | *Stenotrophomonas sp.* | 1032 | OF | A | MK638450 |
| PN2-B10P3-3 | May 2015 | *Stenotrophomonas sp.* | 1034 | OF | A | MK638451 |
| PN2-B10P3-5 | May 2015 | *Pseudomonas sp.* | 1032 | OF | A | MK638013 |
| PN2-B10P3-6 | May 2015 | *Pseudomonas sp.* | 1032 | OF | A | MK638014 |
| PN2-B10P3-7 | May 2015 | *Pseudomonas sp.* | 1032 | OF | A | MK638015 |
| PN2-B10P3-8 | May 2015 | *Pseudomonas sp.* | 1032 | OF | A | MK638016 |
| PN2-B10P3-9 | May 2015 | *Pseudomonas sp.* | 1032 | OF | A | MK638017 |
| PN2-B10P3-10 | May 2015 | *Variovorax sp.* | 464 | OF | A | MK638661 |
| PN2-B10P3-11 | May 2015 | *Variovorax sp.* | 470 | OF | A | MK638662 |
| PN2-B10P3-12 | May 2015 | *Variovorax sp.* | 751 | OF | A | MK638663 |
| PN2-B10P3-13 | May 2015 | *Stenotrophomonas sp.* | 1034 | OF | A | MK638448 |
| PN2-B10P3-14 | May 2015 | *Variovorax sp.* | 506 | OF | A | MK638664 |
| PN2-B10P3-15 | May 2015 | *Mesorhizobium sp.* | 1037 | OF | A | MK638183 |
| PN2-B10P3-17 | May 2015 | *Streptomyces sp.* | 1025 | OF | A | MK638613 |
| PN2-B10P3-18 | May 2015 | *Streptomyces sp.* | 957 | OF | A | MK638614 |
| PN2-B10P3-20 | May 2015 | *Rhizobium sp.* | 1004 | OF | A | MK638432 |
| PN2-B10P3-21 | May 2015 | *Stenotrophomonas sp.* | 1024 | OF | A | MK638449 |
| PN2-B10P5-1 | May 2015 | *Pseudomonas sp.* | 1032 | CF | A | MK638018 |
| PN2-B10P5-2 | May 2015 | *Flavobacterium sp.* | 1027 | CF | A | MK638133 |
| PN2-B10P5-3 | May 2015 | *Rhizobium sp.* | 1017 | CF | A | MK638433 |
| PN2-B10P5-4 | May 2015 | *Pseudomonas sp.* | 1032 | CF | A | MK638021 |
| PN2-B10P5-5 | May 2015 | *Streptomyces sp.* | 1071 | CF | A | MK638515 |
| PN2-B10P5-6 | May 2015 | *Clavibacter sp.* | 1003 | CF | A | MK638110 |
| PN2-B10P5-8 | May 2015 | *Agrobacterium sp.* | 1008 | CF | A | MK638048 |
| PN2-B10P5-10 | May 2015 | *Pseudomonas sp.* | 1032 | CF | A | MK638019 |
| PN2-B10P5-11 | May 2015 | *Pseudomonas sp.* | 1032 | CF | A | MK638020 |
| PN2-B10P5-12 | May 2015 | *Bacillus sp.* | 1027 | CF | A | MK638062 |
| PN2-B10P5-13 | May 2015 | *Streptomyces sp.* | 1066 | CF | A | MK638455 |
| PN2-B10P5-14 | May 2015 | *Pseudomonas sp.* | 1077 | CF | A | MK637959 |
| PN2-B10P5-15 | May 2015 | *Streptomyces sp.* | 1001 | CF | A | MK638594 |
| PN2-B10P5-16 | May 2015 | *Agrobacterium sp.* | 1068 | CF | A | MK638042 |
| PN2-B10P5-17 | May 2015 | *Agrobacterium sp.* | 1070 | CF | A | MK638041 |
| PN2-B10P5-18 | May 2015 | *Streptomyces sp.* | 1073 | CF | A | MK638478 |
| PN2-B10P5-19 | May 2015 | *Streptomyces sp.* | 1069 | CF | A | MK638560 |
| PN2-B10P5-20 | May 2015 | *Rhizobium sp.* | 1075 | CF | A | MK638398 |
| PN2-B10P5-21 | May 2015 | *Phyllobacterium sp.* | 1070 | CF | A | MK638209 |
| PN2-B10P5-22 | May 2015 | *Leifsonia sp.* | 1082 | CF | A | MK638143 |
| PN3-B01P2-1 | July 2015 | *Phyllobacterium sp.* | 998 | CF | A | MK638236 |
| PN3-B01P2-2 | July 2015 | *Streptomyces sp.* | 1018 | CF | A | MK638483 |
| PN3-B01P2-3 | July 2015 | *Rhizobium sp.* | 1023 | CF | A | MK638410 |
| PN3-B01P2-4 | July 2015 | *Mesorhizobium sp.* | 1025 | CF | A | MK638169 |
| PN3-B01P2-5 | July 2015 | *Streptomyces sp.* | 1047 | CF | A | MK638559 |
| PN3-B01P2-6 | July 2015 | *Rhizobium sp.* | 1026 | CF | A | MK638412 |
| PN3-B01P2-7 | July 2015 | *Streptomyces sp.* | 1008 | CF | A | MK638514 |
| PN3-B01P2-9 | July 2015 | *Rhizobium sp.* | 1009 | CF | A | MK638408 |
| PN3-B01P2-10 | July 2015 | *Mesorhizobium sp.* | 1034 | CF | A | MK638167 |
| PN3-B01P2-12 | July 2015 | *Rhizobium sp.* | 1029 | CF | A | MK638411 |
| PN3-B01P2-14 | July 2015 | *Mesorhizobium sp.* | 1026 | CF | A | MK638168 |
| PN3-B01P2-15 | July 2015 | *Leifsonia sp.* | 1043 | CF | A | MK638144 |
| PN3-B01P2-17 | July 2015 | *Rhizobium sp.* | 1020 | CF | A | MK638407 |
| PN3-B01P2-18 | July 2015 | *Inquilinus sp.* | 952 | CF | A | MK638135 |
| PN3-B01P2-19 | July 2015 | *Streptomyces sp.* | 1018 | CF | A | MK638512 |
| PN3-B01P2-20 | July 2015 | *Inquilinus sp.* | 999 | CF | A | MK638136 |
| PN3-B01P2-21 | July 2015 | *Phyllobacterium sp.* | 997 | CF | A | MK638241 |
| PN3-B01P2-22 | July 2015 | *Phyllobacterium sp.* | 1025 | CF | A | MK638239 |
| PN3-B01P2-23 | July 2015 | *Leifsonia sp.* | 1032 | CF | A | MK638145 |
| PN3-B01P4-1 | July 2015 | *Streptomyces sp.* | 1026 | OF | A | MK638563 |
| PN3-B01P4-3 | July 2015 | *Streptomyces sp.* | 1010 | OF | A | MK638558 |
| PN3-B01P4-5 | July 2015 | *Streptomyces sp.* | 1035 | OF | A | MK638562 |
| PN3-B01P4-6 | July 2015 | *Burkholderia sp.* | 926 | OF | A | MK638088 |
| PN3-B01P4-7 | July 2015 | *Streptomyces sp.* | 1015 | OF | A | MK638540 |
| PN3-B01P4-8 | July 2015 | *Phyllobacterium sp.* | 1026 | OF | A | MK638242 |
| PN3-B01P4-9 | July 2015 | *Agrobacterium sp.* | 943 | OF | A | MK638023 |
| PN3-B01P4-10 | July 2015 | *Ensifer sp.* | 1016 | OF | A | MK638119 |
| PN3-B01P4-11 | July 2015 | *Streptomyces sp.* | 1041 | OF | A | MK638518 |
| PN3-B01P4-12 | July 2015 | *Streptomyces sp.* | 1064 | OF | A | MK638509 |
| PN3-B01P4-13 | July 2015 | *Agrobacterium sp.* | 1042 | OF | A | MK638039 |
| PN3-B01P4-14 | July 2015 | *Streptomyces sp.* | 1038 | OF | A | MK638534 |
| PN3-B01P4-15 | July 2015 | *Phyllobacterium sp.* | 1026 | OF | A | MK638237 |
| PN3-B01P4-16 | July 2015 | *Streptomyces sp.* | 1028 | OF | A | MK638516 |
| PN3-B01P4-17 | July 2015 | *Agrobacterium sp.* | 948 | OF | A | MK638024 |
| PN3-B01P4-18 | July 2015 | *Rhizobium sp.* | 1028 | OF | A | MK638409 |
| PN3-B01P4-19 | July 2015 | *Rhizobium sp.* | 818 | OF | A | MK638413 |
| PN3-B01P4-20 | July 2015 | *Rhizobium sp.* | 895 | OF | A | MK638414 |
| PN3-B01P4-21 | July 2015 | *Ensifer sp.* | 1029 | OF | A | MK638120 |
| PN3-B01P4-22 | July 2015 | *Ensifer sp.* | 1023 | OF | A | MK638122 |
| PN3-B01P4-23 | July 2015 | *Rhizobium sp.* | 1039 | OF | A | MK638405 |
| PN3-B01P4-24 | July 2015 | *Streptomyces sp.* | 1025 | OF | A | MK638517 |
| PN3-B02P3-1 | July 2015 | *Phyllobacterium sp.* | 1026 | OF | F | MK638246 |
| PN3-B02P3-2 | July 2015 | *Variovorax sp.* | 266 | OF | F | MK638660 |
| PN3-B02P3-3 | July 2015 | *Pseudomonas sp.* | 951 | OF | F | MK637991 |
| PN3-B02P3-4 | July 2015 | *Phyllobacterium sp.* | 950 | OF | F | MK638238 |
| PN3-B02P3-5 | July 2015 | *Streptomyces sp.* | 1013 | OF | F | MK638600 |
| PN3-B02P3-6 | July 2015 | *Bacillus sp.* | 841 | OF | F | MK638072 |
| PN3-B02P3-7 | July 2015 | *Bacillus sp.* | 982 | OF | F | MK638074 |
| PN3-B02P3-8 | July 2015 | *Bacillus sp.* | 1024 | OF | F | MK638075 |
| PN3-B02P3-9 | July 2015 | *Bacillus sp.* | 1009 | OF | F | MK638079 |
| PN3-B02P3-10 | July 2015 | *Bacillus sp.* | 1011 | OF | F | MK638077 |
| PN3-B02P3-11 | July 2015 | *Bacillus sp.* | 967 | OF | F | MK638073 |
| PN3-B02P3-12 | July 2015 | *Bacillus sp.* | 1024 | OF | F | MK638076 |
| PN3-B02P3-13 | July 2015 | *Streptomyces sp.* | 1010 | OF | F | MK638526 |
| PN3-B02P3-14 | July 2015 | *Streptomyces sp.* | 1031 | OF | F | MK638510 |
| PN3-B02P3-15 | July 2015 | *Bacillus sp.* | 977 | OF | F | MK638078 |
| PN3-B02P3-17 | July 2015 | *Streptomyces sp.* | 1018 | OF | F | MK638523 |
| PN3-B02P3-18 | July 2015 | *Phyllobacterium sp.* | 999 | OF | F | MK638243 |
| PN3-B02P3-19 | July 2015 | *Phyllobacterium sp.* | 1026 | OF | F | MK638244 |
| PN3-B02P3-20 | July 2015 | *Phyllobacterium sp.* | 1024 | OF | F | MK638245 |
| PN3-B02P3-21 | July 2015 | *Streptomyces sp.* | 1018 | OF | F | MK638535 |
| PN3-B02P3-22 | July 2015 | *Bradyrhizobium sp.* | 1027 | OF | F | MK638083 |
| PN3-B02P4-1 | July 2015 | *Phyllobacterium sp.* | 1034 | CF | F | MK638235 |
| PN3-B02P4-3 | July 2015 | *Mesorhizobium sp.* | 1026 | CF | F | MK638166 |
| PN3-B02P4-4 | July 2015 | *Phyllobacterium sp.* | 907 | CF | F | MK638247 |
| PN3-B02P4-5 | July 2015 | *Herbiconiux sp.* | 293 | CF | F | MK638668 |
| PN3-B02P4-6 | July 2015 | *Phyllobacterium sp.* | 663 | CF | F | MK638248 |
| PN3-B02P4-7 | July 2015 | *Phyllobacterium sp.* | 1030 | CF | F | MK638240 |
| PN3-B02P4-15 | July 2015 | *Streptomyces sp.* | 1057 | CF | F | MK638561 |
| PN3-B02P4-17 | July 2015 | *Streptomyces sp.* | 1012 | CF | F | MK638601 |
| PN3-B02P4-18 | July 2015 | *Streptomyces sp.* | 914 | CF | F | MK638603 |
| PN3-B02P4-19 | July 2015 | *Phyllobacterium sp.* | 1024 | CF | F | MK638234 |
| PN3-B02P4-20 | July 2015 | *Pseudaminobacter sp.* | 1012 | CF | F | MK638382 |
| PN3-B02P4-21 | July 2015 | *Streptomyces sp.* | 1018 | CF | F | MK638513 |
| PN3-B02P4-22 | July 2015 | *Rhizobium sp.* | 1023 | CF | F | MK638406 |
| PN3-B03P1-1 | July 2015 | *Agrobacterium sp.* | 1029 | CF | A | MK638038 |
| PN3-B03P1-2 | July 2015 | *Variovorax sp.* | 624 | CF | A | MK638659 |
| PN3-B03P1-3 | July 2015 | *Mesorhizobium sp.* | 1001 | CF | A | MK638171 |
| PN3-B03P1-4 | July 2015 | *Streptomyces sp.* | 814 | CF | A | MK638602 |
| PN3-B03P1-5 | July 2015 | *Mesorhizobium sp.* | 1052 | CF | A | MK638170 |
| PN3-B03P1-6 | July 2015 | *Ensifer sp.* | 1021 | CF | A | MK638121 |
| PN3-B03P1-7 | July 2015 | *Streptomyces sp.* | 1022 | CF | A | MK638564 |
| PN3-B03P1-10 | July 2015 | *Streptomyces sp.* | 908 | CF | A | MK638604 |
| PN3-B03P1-12 | July 2015 | *Inquilinus sp.* | 1019 | CF | A | MK638137 |
| PN3-B03P1-13 | July 2015 | *Phyllobacterium sp.* | 1011 | CF | A | MK638233 |
| PN3-B03P1-14 | July 2015 | *Leifsonia sp.* | 1019 | CF | A | MK638146 |
| PN3-B03P1-16 | July 2015 | *Streptomyces sp.* | 1010 | CF | A | MK638599 |
| PN3-B03P1-17 | July 2015 | *Streptomyces sp.* | 1025 | CF | A | MK638511 |
| PN3-B03P1-18 | July 2015 | *Dyella sp.* | 962 | CF | A | MK638117 |
| PN3-B03P1-19 | July 2015 | *Streptomyces sp.* | 1010 | CF | A | MK638533 |
| PN3-B03P1-20 | July 2015 | *Variovorax sp.* | 991 | CF | A | MK638656 |
| PN3-B03P1-21 | July 2015 | *Streptomyces sp.* | 1060 | CF | A | MK638550 |
| PN3-B03P1-22 | July 2015 | *Streptomyces sp.* | 1034 | CF | A | MK638520 |
| PN3-B03P1-23 | July 2015 | *Streptomyces sp.* | 1024 | CF | A | MK638525 |
| PN3-B03P2-1 | July 2015 | *Dyella sp.* | 1051 | OF | A | MK638665 |
| PN3-B03P2-2 | July 2015 | *Dyella sp.* | 1039 | OF | A | MK638666 |
| PN3-B03P2-3 | July 2015 | *Streptomyces sp.* | 1070 | OF | A | MK638581 |
| PN3-B03P2-4 | July 2015 | *Streptomyces sp.* | 1019 | OF | A | MK638548 |
| PN3-B03P2-5 | July 2015 | *Streptomyces sp.* | 1059 | OF | A | MK638547 |
| PN3-B03P2-6 | July 2015 | *Rhizobium sp.* | 1050 | OF | A | MK638420 |
| PN3-B03P2-7 | July 2015 | *Streptomyces sp.* | 1070 | OF | A | MK638546 |
| PN3-B03P2-8 | July 2015 | *Rhizobium sp.* | 1062 | OF | A | MK638415 |
| PN3-B03P2-9 | July 2015 | *Streptomyces sp.* | 1066 | OF | A | MK638544 |
| PN3-B03P2-10 | July 2015 | *Rhizobium sp.* | 1058 | OF | A | MK638418 |
| PN3-B03P2-12 | July 2015 | *Leifsonia sp.* | 1058 | OF | A | MK638147 |
| PN3-B03P2-13 | July 2015 | *Streptomyces sp.* | 1041 | OF | A | MK638579 |
| PN3-B03P2-14 | July 2015 | *Mesorhizobium sp.* | 1061 | OF | A | MK638172 |
| PN3-B03P2-15 | July 2015 | *Mesorhizobium sp.* | 1073 | OF | A | MK638173 |
| PN3-B03P2-16 | July 2015 | *Streptomyces sp.* | 1002 | OF | A | MK638586 |
| PN3-B03P2-17 | July 2015 | *Streptomyces sp.* | 1022 | OF | A | MK638543 |
| PN3-B03P2-18 | July 2015 | *Streptomyces sp.* | 1018 | OF | A | MK638625 |
| PN3-B03P2-19 | July 2015 | *Pseudomonas sp.* | 1069 | OF | A | MK637995 |
| PN3-B03P2-20 | July 2015 | *Luteibacter sp.* | 1039 | OF | A | MK638152 |
| PN3-B03P2-21 | July 2015 | *Streptomyces sp.* | 1070 | OF | A | MK638552 |
| PN3-B03P2-22 | July 2015 | *Streptomyces sp.* | 1024 | OF | A | MK638609 |
| PN3-B04P1-1 | July 2015 | *Pseudomonas sp.* | 1067 | CF | F | MK637996 |
| PN3-B04P1-2 | July 2015 | *Streptomyces sp.* | 1055 | CF | F | MK638577 |
| PN3-B04P1-3 | July 2015 | *Rhizobium sp.* | 1069 | CF | F | MK638421 |
| PN3-B04P1-4 | July 2015 | *Phyllobacterium sp.* | 1052 | CF | F | MK638259 |
| PN3-B04P1-6 | July 2015 | *Rhizobium sp.* | 1024 | CF | F | MK638422 |
| PN3-B04P1-7 | July 2015 | *Phyllobacterium sp.* | 1061 | CF | F | MK638249 |
| PN3-B04P1-8 | July 2015 | *Microbacterium sp.* | 1048 | CF | F | MK638188 |
| PN3-B04P1-9 | July 2015 | *Stenotrophomonas sp.* | 1041 | CF | F | MK638446 |
| PN3-B04P1-10 | July 2015 | *Mesorhizobium sp.* | 1042 | CF | F | MK638175 |
| PN3-B04P1-11 | July 2015 | *Streptomyces sp.* | 1055 | CF | F | MK638578 |
| PN3-B04P1-12 | July 2015 | *Phyllobacterium sp.* | 1049 | CF | F | MK638250 |
| PN3-B04P1-14 | July 2015 | *Phyllobacterium sp.* | 1068 | CF | F | MK638256 |
| PN3-B04P1-15 | July 2015 | *Streptomyces sp.* | 1073 | CF | F | MK638551 |
| PN3-B04P1-17 | July 2015 | *Microbacterium sp.* | 1047 | CF | F | MK638187 |
| PN3-B04P1-18 | July 2015 | *Tardiphaga sp.* | 1035 | CF | F | MK638649 |
| PN3-B04P1-20 | July 2015 | *Caulobacter sp.* | 541 | CF | F | MK638105 |
| PN3-B04P1-21 | July 2015 | *Streptomyces sp.* | 1024 | CF | F | MK638589 |
| PN3-B04P1-22 | July 2015 | *Streptomyces sp.* | 1007 | CF | F | MK638554 |
| PN3-B04P2-1 | July 2015 | *Luteibacter sp.* | 1029 | OF | F | MK638151 |
| PN3-B04P2-2 | July 2015 | *Pseudomonas sp.* | 1055 | OF | F | MK637993 |
| PN3-B04P2-3 | July 2015 | *Streptomyces sp.* | 1061 | OF | F | MK638580 |
| PN3-B04P2-4 | July 2015 | *Pseudomonas sp.* | 1055 | OF | F | MK637997 |
| PN3-B04P2-5 | July 2015 | *Streptomyces sp.* | 1061 | OF | F | MK638582 |
| PN3-B04P2-6 | July 2015 | *Rhizobium sp.* | 1026 | OF | F | MK638419 |
| PN3-B04P2-7 | July 2015 | *Phyllobacterium sp.* | 1038 | OF | F | MK638260 |
| PN3-B04P2-8 | July 2015 | *Ensifer sp.* | 1029 | OF | F | MK638123 |
| PN3-B04P2-9 | July 2015 | *Mycobacterium sp.* | 1067 | OF | F | MK638195 |
| PN3-B04P2-10 | July 2015 | *Luteibacter sp.* | 1051 | OF | F | MK638150 |
| PN3-B04P2-11 | July 2015 | *Agrobacterium sp.* | 1065 | OF | F | MK637994 |
| PN3-B04P2-12 | July 2015 | *Mycobacterium sp.* | 1047 | OF | F | MK638194 |
| PN3-B04P2-13 | July 2015 | *Phyllobacterium sp.* | 541 | OF | F | MK638367 |
| PN3-B04P2-14 | July 2015 | *Streptomyces sp.* | 1018 | OF | F | MK638626 |
| PN3-B04P2-15 | July 2015 | *Streptomyces sp.* | 955 | OF | F | MK638607 |
| PN3-B04P2-16 | July 2015 | *Rhizobium sp.* | 1064 | OF | F | MK638416 |
| PN3-B04P2-17 | July 2015 | *Mesorhizobium sp.* | 1057 | OF | F | MK638174 |
| PN3-B04P2-19 | July 2015 | *Mesorhizobium sp.* | 1068 | OF | F | MK638177 |
| PN3-B04P2-20 | July 2015 | *Streptomyces sp.* | 1055 | OF | F | MK638605 |
| PN3-B04P2-21 | July 2015 | *Rhizobium sp.* | 1062 | OF | F | MK638417 |
| PN3-B04P2-22 | July 2015 | *Streptomyces sp.* | 1067 | OF | F | MK638584 |
| PN3-B04P2-23 | July 2015 | *Phyllobacterium sp.* | 541 | OF | F | MK638366 |
| PN3-B05P1-2 | July 2015 | *Phyllobacterium sp.* | 1051 | OF | A | MK638255 |
| PN3-B05P1-3 | July 2015 | *Inquilinus sp.* | 1053 | OF | A | MK638138 |
| PN3-B05P1-4 | July 2015 | *Microbacterium sp.* | 507 | OF | A | MK638191 |
| PN3-B05P1-5 | July 2015 | *Phyllobacterium sp.* | 1073 | OF | A | MK638251 |
| PN3-B05P1-6 | July 2015 | *Streptomyces sp.* | 1025 | OF | A | MK638588 |
| PN3-B05P1-7 | July 2015 | *Paraburkholderia sp.* | 1036 | OF | A | MK638089 |
| PN3-B05P1-8 | July 2015 | *Inquilinus sp.* | 1047 | OF | A | MK638139 |
| PN3-B05P1-9 | July 2015 | *Streptomyces sp.* | 1073 | OF | A | MK638583 |
| PN3-B05P1-10 | July 2015 | *Streptomyces sp.* | 891 | OF | A | MK638608 |
| PN3-B05P1-11 | July 2015 | *Phyllobacterium sp.* | 1029 | OF | A | MK638258 |
| PN3-B05P1-12 | July 2015 | *Phyllobacterium sp.* | 1070 | OF | A | MK638257 |
| PN3-B05P1-13 | July 2015 | *Pseudomonas sp.* | 1030 | OF | A | MK637998 |
| PN3-B05P1-14 | July 2015 | *Streptomyces sp.* | 1022 | OF | A | MK638585 |
| PN3-B05P1-15 | July 2015 | *Mesorhizobium sp.* | 1074 | OF | A | MK638176 |
| PN3-B05P1-16 | July 2015 | *Streptomyces sp.* | 864 | OF | A | MK638606 |
| PN3-B05P1-17 | July 2015 | *Phyllobacterium sp.* | 1043 | OF | A | MK638254 |
| PN3-B05P1-18 | July 2015 | *Streptomyces sp.* | 1055 | OF | A | MK638587 |
| PN3-B05P1-19 | July 2015 | *Rhizobium sp.* | 1026 | OF | A | MK638423 |
| PN3-B05P1-21 | July 2015 | *Agrobacterium sp.* | 1062 | OF | A | MK638025 |
| PN3-B05P3-1 | July 2015 | *Phyllobacterium sp.* | 1052 | CF | A | MK638253 |
| PN3-B05P3-2 | July 2015 | *Streptomyces sp.* | 1060 | CF | A | MK638524 |
| PN3-B05P3-3 | July 2015 | *Agromyces sp.* | 1023 | CF | A | MK638051 |
| PN3-B05P3-4 | July 2015 | *Streptomyces sp.* | 1054 | CF | A | MK638527 |
| PN3-B05P3-5 | July 2015 | *Pseudomonas sp.* | 1067 | CF | A | MK637992 |
| PN3-B05P3-6 | July 2015 | *Phyllobacterium sp.* | 1065 | CF | A | MK638252 |
| PN3-B05P3-7 | July 2015 | *Phyllobacterium sp.* | 1031 | CF | A | MK638261 |
| PN3-B05P3-8 | July 2015 | *Phyllobacterium sp.* | 1055 | CF | A | MK638262 |
| PN3-B05P3-9 | July 2015 | *Phyllobacterium sp.* | 1051 | CF | A | MK638272 |
| PN3-B05P3-10 | July 2015 | *Streptomyces sp.* | 931 | CF | A | MK638484 |
| PN3-B05P3-11 | July 2015 | *Inquilinus sp.* | 1054 | CF | A | MK638140 |
| PN3-B05P3-12 | July 2015 | *Phyllobacterium sp.* | 1053 | CF | A | MK638285 |
| PN3-B05P3-13 | July 2015 | *Pseudomonas sp.* | 1074 | CF | A | MK638000 |
| PN3-B05P3-14 | July 2015 | *Phyllobacterium sp.* | 1052 | CF | A | MK638264 |
| PN3-B05P3-15 | July 2015 | *Phyllobacterium sp.* | 1053 | CF | A | MK638288 |
| PN3-B05P3-17 | July 2015 | *Streptomyces sp.* | 1060 | CF | A | MK638490 |
| PN3-B05P3-18 | July 2015 | *Phyllobacterium sp.* | 1062 | CF | A | MK638269 |
| PN3-B05P3-19 | July 2015 | *Phyllobacterium sp.* | 1034 | CF | A | MK638277 |
| PN3-B05P3-20 | July 2015 | *Phyllobacterium sp.* | 1045 | CF | A | MK638270 |
| PN3-B05P3-21 | July 2015 | *Streptomyces sp.* | 1064 | CF | A | MK638491 |
| PN3-B05P3-23 | July 2015 | *Streptomyces sp.* | 1060 | CF | A | MK638508 |
| PN3-B05P3-24 | July 2015 | *Streptomyces sp.* | 1060 | CF | A | MK638610 |
| PN3-B06P2-1 | July 2015 | *Phyllobacterium sp.* | 588 | CF | F | MK638336 |
| PN3-B06P2-2 | July 2015 | *Phyllobacterium sp.* | 1029 | CF | F | MK638296 |
| PN3-B06P2-3 | July 2015 | *Phyllobacterium sp.* | 1004 | CF | F | MK638309 |
| PN3-B06P2-4 | July 2015 | *Phyllobacterium sp.* | 1034 | CF | F | MK638298 |
| PN3-B06P2-5 | July 2015 | *Phyllobacterium sp.* | 1065 | CF | F | MK638276 |
| PN3-B06P2-6 | July 2015 | *Phyllobacterium sp.* | 1035 | CF | F | MK638291 |
| PN3-B06P2-7 | July 2015 | *Devosia sp.* | 1030 | CF | F | MK638111 |
| PN3-B06P2-8 | July 2015 | *Phyllobacterium sp.* | 1062 | CF | F | MK638275 |
| PN3-B06P2-9 | July 2015 | *Phyllobacterium sp.* | 1062 | CF | F | MK638273 |
| PN3-B06P2-10 | July 2015 | *Streptomyces sp.* | 1060 | CF | F | MK638505 |
| PN3-B06P2-11 | July 2015 | *Phyllobacterium sp.* | 1053 | CF | F | MK638265 |
| PN3-B06P2-12 | July 2015 | *Phyllobacterium sp.* | 837 | CF | F | MK638297 |
| PN3-B06P2-13 | July 2015 | *Bacillus sp.* | 527 | CF | F | MK638080 |
| PN3-B06P2-14 | July 2015 | *Phyllobacterium sp.* | 1065 | CF | F | MK638284 |
| PN3-B06P2-15 | July 2015 | *Phyllobacterium sp.* | 1053 | CF | F | MK638266 |
| PN3-B06P2-16 | July 2015 | *Streptomyces sp.* | 1068 | CF | F | MK638507 |
| PN3-B06P2-17 | July 2015 | *Streptomyces sp.* | 605 | CF | F | MK638627 |
| PN3-B06P2-18 | July 2015 | *Rhizobium sp.* | 939 | CF | F | MK638424 |
| PN3-B06P2-19 | July 2015 | *Phyllobacterium sp.* | 1030 | CF | F | MK638274 |
| PN3-B06P2-20 | July 2015 | *Streptomyces sp.* | 1064 | CF | F | MK638489 |
| PN3-B06P2-21 | July 2015 | *Phyllobacterium sp.* | 1073 | CF | F | MK638271 |
| PN3-B06P2-22 | July 2015 | *Chryseobacterium sp.* | 1045 | CF | F | MK638109 |
| PN3-B06P2-23 | July 2015 | *Streptomyces sp.* | 1062 | CF | F | MK638492 |
| PN3-B06P4-1 | July 2015 | *Pseudomonas sp.* | 1030 | OF | F | MK638002 |
| PN3-B06P4-2 | July 2015 | *Pseudomonas sp.* | 1068 | OF | F | MK637999 |
| PN3-B06P4-3 | July 2015 | *Phyllobacterium sp.* | 1055 | OF | F | MK638279 |
| PN3-B06P4-4 | July 2015 | *Variovorax sp.* | 1031 | OF | F | MK638657 |
| PN3-B06P4-5 | July 2015 | *Streptomyces sp.* | 1060 | OF | F | MK638538 |
| PN3-B06P4-6 | July 2015 | *Phyllobacterium sp.* | 1070 | OF | F | MK638263 |
| PN3-B06P4-7 | July 2015 | *Phyllobacterium sp.* | 1050 | OF | F | MK638287 |
| PN3-B06P4-8 | July 2015 | *Phyllobacterium sp.* | 1065 | OF | F | MK638282 |
| PN3-B06P4-9 | July 2015 | *Phyllobacterium sp.* | 1029 | OF | F | MK638278 |
| PN3-B06P4-10 | July 2015 | *Streptomyces sp.* | 1060 | OF | F | MK638496 |
| PN3-B06P4-11 | July 2015 | *Streptomyces sp.* | 1059 | OF | F | MK638506 |
| PN3-B06P4-12 | July 2015 | *Phyllobacterium sp.* | 1029 | OF | F | MK638302 |
| PN3-B06P4-13 | July 2015 | *Phyllobacterium sp.* | 1031 | OF | F | MK638294 |
| PN3-B06P4-14 | July 2015 | *Streptomyces sp.* | 1064 | OF | F | MK638493 |
| PN3-B06P4-15 | July 2015 | *Streptomyces sp.* | 1055 | OF | F | MK638494 |
| PN3-B06P4-16 | July 2015 | *Phyllobacterium sp.* | 1055 | OF | F | MK638268 |
| PN3-B06P4-17 | July 2015 | *Streptomyces sp.* | 1056 | OF | F | MK638590 |
| PN3-B06P4-18 | July 2015 | *Streptomyces sp.* | 1025 | OF | F | MK638498 |
| PN3-B06P4-19 | July 2015 | *Streptomyces sp.* | 1062 | OF | F | MK638499 |
| PN3-B06P4-20 | July 2015 | *Phyllobacterium sp.* | 1055 | OF | F | MK638290 |
| PN3-B06P4-21 | July 2015 | *Phyllobacterium sp.* | 1061 | OF | F | MK638267 |
| PN3-B06P4-22 | July 2015 | *Phyllobacterium sp.* | 1037 | OF | F | MK638303 |
| PN3-B06P4-23 | July 2015 | *Streptomyces sp.* | 1056 | OF | F | MK638500 |
| PN3-B07P1-1 | July 2015 | *Streptomyces sp.* | 1073 | OF | F | MK638497 |
| PN3-B07P1-3 | July 2015 | *Phyllobacterium sp.* | 1061 | OF | F | MK638280 |
| PN3-B07P1-4 | July 2015 | *Phyllobacterium sp.* | 1027 | OF | F | MK638293 |
| PN3-B07P1-5 | July 2015 | *Phyllobacterium sp.* | 1034 | OF | F | MK638306 |
| PN3-B07P1-6 | July 2015 | *Phyllobacterium sp.* | 1062 | OF | F | MK638286 |
| PN3-B07P1-7 | July 2015 | *Phyllobacterium sp.* | 1006 | OF | F | MK638310 |
| PN3-B07P1-8 | July 2015 | *Phyllobacterium sp.* | 1033 | OF | F | MK638301 |
| PN3-B07P1-9 | July 2015 | *Streptomyces sp.* | 1064 | OF | F | MK638549 |
| PN3-B07P1-11 | July 2015 | *Pseudomonas sp.* | 900 | OF | F | MK638011 |
| PN3-B07P1-12 | July 2015 | *Streptomyces sp.* | 1056 | OF | F | MK638591 |
| PN3-B07P1-13 | July 2015 | *Phyllobacterium sp.* | 1066 | OF | F | MK638289 |
| PN3-B07P1-14 | July 2015 | *Phyllobacterium sp.* | 1035 | OF | F | MK638307 |
| PN3-B07P1-15 | July 2015 | *Streptomyces sp.* | 1053 | OF | F | MK638542 |
| PN3-B07P1-16 | July 2015 | *Streptomyces sp.* | 1046 | OF | F | MK638487 |
| PN3-B07P1-17 | July 2015 | *Phyllobacterium sp.* | 1046 | OF | F | MK638292 |
| PN3-B07P1-18 | July 2015 | *Streptomyces sp.* | 1056 | OF | F | MK638541 |
| PN3-B07P1-19 | July 2015 | *Phyllobacterium sp.* | 999 | OF | F | MK638295 |
| PN3-B07P1-20 | July 2015 | *Phyllobacterium sp.* | 1054 | OF | F | MK638281 |
| PN3-B07P1-21 | July 2015 | *Phyllobacterium sp.* | 999 | OF | F | MK638300 |
| PN3-B07P1-22 | July 2015 | *Phyllobacterium sp.* | 1035 | OF | F | MK638299 |
| PN3-B07P5-1 | July 2015 | *Phyllobacterium sp.* | 1030 | CF | F | MK638283 |
| PN3-B07P5-2 | July 2015 | *Phyllobacterium sp.* | 1021 | CF | F | MK638376 |
| PN3-B07P5-3 | July 2015 | *Phyllobacterium sp.* | 1006 | CF | F | MK638311 |
| PN3-B07P5-4 | July 2015 | *Phyllobacterium sp.* | 1010 | CF | F | MK638305 |
| PN3-B07P5-5 | July 2015 | *Agrobacterium sp.* | 946 | CF | F | MK638037 |
| PN3-B07P5-6 | July 2015 | *Mesorhizobium sp.* | 1027 | CF | F | MK638178 |
| PN3-B07P5-7 | July 2015 | *Mesorhizobium sp.* | 1030 | CF | F | MK638179 |
| PN3-B07P5-8 | July 2015 | *Streptomyces sp.* | 1064 | CF | F | MK638495 |
| PN3-B07P5-9 | July 2015 | *Streptomyces sp.* | 1018 | CF | F | MK638630 |
| PN3-B07P5-10 | July 2015 | *Agrobacterium sp.* | 1065 | CF | F | MK638001 |
| PN3-B07P5-11 | July 2015 | *Streptomyces sp.* | 670 | CF | F | MK638628 |
| PN3-B07P5-12 | July 2015 | *Streptomyces sp.* | 1059 | CF | F | MK638488 |
| PN3-B07P5-13 | July 2015 | *Streptomyces sp.* | 1018 | CF | F | MK638629 |
| PN3-B07P5-14 | July 2015 | *Streptomyces sp.* | 1070 | CF | F | MK638501 |
| PN3-B07P5-15 | July 2015 | *Ensifer sp.* | 1072 | CF | F | MK638124 |
| PN3-B07P5-16 | July 2015 | *Mesorhizobium sp.* | 1070 | CF | F | MK638180 |
| PN3-B07P5-18 | July 2015 | *Inquilinus sp.* | 1035 | CF | F | MK638141 |
| PN3-B07P5-19 | July 2015 | *Agrobacterium sp.* | 1030 | CF | F | MK638036 |
| PN3-B07P5-20 | July 2015 | *Phyllobacterium sp.* | 925 | CF | F | MK638314 |
| PN3-B07P5-22 | July 2015 | *Mesorhizobium sp.* | 1072 | CF | F | MK638181 |
| PN3-B07P5-23 | July 2015 | *Pseudomonas sp.* | 1030 | CF | F | MK638006 |
| PN3-B08P4-1 | July 2015 | *Leifsonia sp.* | 1059 | CF | A | MK638148 |
| PN3-B08P4-2 | July 2015 | *Agrobacterium sp.* | 1051 | CF | A | MK638032 |
| PN3-B08P4-3 | July 2015 | *Agrobacterium sp.* | 1070 | CF | A | MK638030 |
| PN3-B08P4-4 | July 2015 | *Agrobacterium sp.* | 1032 | CF | A | MK638035 |
| PN3-B08P4-5 | July 2015 | *Agrobacterium sp.* | 1071 | CF | A | MK638031 |
| PN3-B08P4-6 | July 2015 | *Agrobacterium sp.* | 1029 | CF | A | MK638033 |
| PN3-B08P4-7 | July 2015 | *Phyllobacterium sp.* | 1070 | CF | A | MK638327 |
| PN3-B08P4-9 | July 2015 | *Agrobacterium sp.* | 1068 | CF | A | MK638027 |
| PN3-B08P4-10 | July 2015 | *Agrobacterium sp.* | 1029 | CF | A | MK638029 |
| PN3-B08P4-11 | July 2015 | *Agrobacterium sp.* | 1022 | CF | A | MK638034 |
| PN3-B08P4-12 | July 2015 | *Phyllobacterium sp.* | 1042 | CF | A | MK638338 |
| PN3-B08P4-14 | July 2015 | *Pseudomonas sp.* | 1057 | CF | A | MK638004 |
| PN3-B08P4-15 | July 2015 | *Phyllobacterium sp.* | 1027 | CF | A | MK638370 |
| PN3-B08P4-16 | July 2015 | *Agrobacterium sp.* | 1061 | CF | A | MK638028 |
| PN3-B08P4-18 | July 2015 | *Pseudomonas sp.* | 1085 | CF | A | MK638003 |
| PN3-B08P4-19 | July 2015 | *Phyllobacterium sp.* | 1068 | CF | A | MK638319 |
| PN3-B08P4-21 | July 2015 | *Phyllobacterium sp.* | 1072 | CF | A | MK638347 |
| PN3-B08P4-22 | July 2015 | *Leifsonia sp.* | 1046 | CF | A | MK638149 |
| PN3-B08P5-1 | July 2015 | *Pseudomonas sp.* | 1043 | OF | A | MK638008 |
| PN3-B08P5-2 | July 2015 | *Phyllobacterium sp.* | 1030 | OF | A | MK638334 |
| PN3-B08P5-3 | July 2015 | *Pseudomonas sp.* | 1056 | OF | A | MK638005 |
| PN3-B08P5-4 | July 2015 | *Phyllobacterium sp.* | 1073 | OF | A | MK638340 |
| PN3-B08P5-5 | July 2015 | *Phyllobacterium sp.* | 1068 | OF | A | MK638320 |
| PN3-B08P5-6 | July 2015 | *Streptomyces sp.* | 1070 | OF | A | MK638502 |
| PN3-B08P5-7 | July 2015 | *Pseudomonas sp.* | 1043 | OF | A | MK638007 |
| PN3-B08P5-8 | July 2015 | *Phyllobacterium sp.* | 1068 | OF | A | MK638329 |
| PN3-B08P5-9 | July 2015 | *Agrobacterium sp.* | 1072 | OF | A | MK638026 |
| PN3-B08P5-10 | July 2015 | *Phyllobacterium sp.* | 1034 | OF | A | MK638352 |
| PN3-B08P5-11 | July 2015 | *Microbacterium sp.* | 855 | OF | A | MK638192 |
| PN3-B08P5-12 | July 2015 | *Streptomyces sp.* | 1018 | OF | A | MK638631 |
| PN3-B08P5-13 | July 2015 | *Phyllobacterium sp.* | 1054 | OF | A | MK638331 |
| PN3-B08P5-14 | July 2015 | *Streptomyces sp.* | 955 | OF | A | MK638632 |
| PN3-B08P5-15 | July 2015 | *Phyllobacterium sp.* | 1070 | OF | A | MK638426 |
| PN3-B08P5-16 | July 2015 | *Streptomyces sp.* | 561 | OF | A | MK638633 |
| PN3-B08P5-18 | July 2015 | *Phyllobacterium sp.* | 1042 | OF | A | MK638346 |
| PN3-B08P5-19 | July 2015 | *Phyllobacterium sp.* | 1070 | OF | A | MK638428 |
| PN3-B08P5-20 | July 2015 | *Rhizobium sp.* | 1070 | OF | A | MK638430 |
| PN3-B08P5-21 | July 2015 | *Phyllobacterium sp.* | 1068 | OF | A | MK638321 |
| PN3-B08P5-22 | July 2015 | *Streptomyces sp.* | 1071 | OF | A | MK638504 |
| PN3-B08P5-23 | July 2015 | *Stenotrophomonas sp.* | 1039 | OF | A | MK638447 |
| PN3-B08P5-24 | July 2015 | *Streptomyces sp.* | 955 | OF | A | MK638634 |
| PN3-B09P3-2 | July 2015 | *Ensifer sp.* | 1070 | CF | F | MK638127 |
| PN3-B09P3-3 | July 2015 | *Afipia sp.* | 834 | CF | F | MK638667 |
| PN3-B09P3-4 | July 2015 | *Streptomyces sp.* | 1070 | CF | F | MK638503 |
| PN3-B09P3-5 | July 2015 | *Streptomyces sp.* | 1019 | CF | F | MK638638 |
| PN3-B09P3-6 | July 2015 | *Streptomyces sp.* | 1018 | CF | F | MK638639 |
| PN3-B09P3-8 | July 2015 | *Phyllobacterium sp.* | 1031 | CF | F | MK638317 |
| PN3-B09P3-9 | July 2015 | *Bosea sp.* | 1072 | CF | F | MK638084 |
| PN3-B09P3-10 | July 2015 | *Phyllobacterium sp.* | 1073 | CF | F | MK638425 |
| PN3-B09P3-11 | July 2015 | *Ensifer sp.* | 1070 | CF | F | MK638126 |
| PN3-B09P3-12 | July 2015 | *Streptomyces sp.* | 660 | CF | F | MK638635 |
| PN3-B09P3-14 | July 2015 | *Phyllobacterium sp.* | 1070 | CF | F | MK638335 |
| PN3-B09P3-15 | July 2015 | *Phyllobacterium sp.* | 1029 | CF | F | MK638343 |
| PN3-B09P3-16 | July 2015 | *Phyllobacterium sp.* | 1070 | CF | F | MK638322 |
| PN3-B09P3-17 | July 2015 | *Ensifer sp.* | 1029 | CF | F | MK638128 |
| PN3-B09P3-18 | July 2015 | *Phyllobacterium sp.* | 1052 | CF | F | MK638351 |
| PN3-B09P3-19 | July 2015 | *Streptomyces sp.* | 1018 | CF | F | MK638636 |
| PN3-B09P3-20 | July 2015 | *Streptomyces sp.* | 1018 | CF | F | MK638637 |
| PN3-B09P3-21 | July 2015 | *Phyllobacterium sp.* | 1055 | CF | F | MK638353 |
| PN3-B09P3-22 | July 2015 | *Phyllobacterium sp.* | 1052 | CF | F | MK638350 |
| PN3-B09P5-1 | July 2015 | *Phyllobacterium sp.* | 1068 | OF | F | MK638318 |
| PN3-B09P5-2 | July 2015 | *Phyllobacterium sp.* | 1070 | OF | F | MK638330 |
| PN3-B09P5-3 | July 2015 | *Phyllobacterium sp.* | 1062 | OF | F | MK638341 |
| PN3-B09P5-4 | July 2015 | *Phyllobacterium sp.* | 1071 | OF | F | MK638315 |
| PN3-B09P5-5 | July 2015 | *Phyllobacterium sp.* | 1073 | OF | F | MK638324 |
| PN3-B09P5-6 | July 2015 | *Phyllobacterium sp.* | 1025 | OF | F | MK638371 |
| PN3-B09P5-7 | July 2015 | *Phyllobacterium sp.* | 1011 | OF | F | MK638373 |
| PN3-B09P5-8 | July 2015 | *Streptomyces sp.* | 393 | OF | F | MK638643 |
| PN3-B09P5-9 | July 2015 | *Streptomyces sp.* | 1018 | OF | F | MK638644 |
| PN3-B09P5-10 | July 2015 | *Paenarthrobacter sp.* | 1040 | OF | F | MK638200 |
| PN3-B09P5-11 | July 2015 | *Phyllobacterium sp.* | 944 | OF | F | MK638357 |
| PN3-B09P5-12 | July 2015 | *Phyllobacterium sp.* | 1070 | OF | F | MK638328 |
| PN3-B09P5-13 | July 2015 | *Phyllobacterium sp.* | 1070 | OF | F | MK638427 |
| PN3-B09P5-14 | July 2015 | *Streptomyces sp.* | 1018 | OF | F | MK638640 |
| PN3-B09P5-15 | July 2015 | *Streptomyces sp.* | 1018 | OF | F | MK638641 |
| PN3-B09P5-16 | July 2015 | *Phyllobacterium sp.* | 1034 | OF | F | MK638356 |
| PN3-B09P5-17 | July 2015 | *Phyllobacterium sp.* | 1070 | OF | F | MK638431 |
| PN3-B09P5-18 | July 2015 | *Streptomyces sp.* | 601 | OF | F | MK638642 |
| PN3-B09P5-19 | July 2015 | *Phyllobacterium sp.* | 1034 | OF | F | MK638354 |
| PN3-B09P5-20 | July 2015 | *Phyllobacterium sp.* | 1026 | OF | F | MK638358 |
| PN3-B09P5-22 | July 2015 | *Phyllobacterium sp.* | 1052 | OF | F | MK638349 |
| PN3-B10P3-1 | July 2015 | *Microbacterium sp.* | 1020 | OF | A | MK638189 |
| PN3-B10P3-2 | July 2015 | *Phyllobacterium sp.* | 1060 | OF | A | MK638344 |
| PN3-B10P3-4 | July 2015 | *Phyllobacterium sp.* | 1070 | OF | A | MK638316 |
| PN3-B10P3-5 | July 2015 | *Agrobacterium sp.* | 1008 | OF | A | MK638049 |
| PN3-B10P3-6 | July 2015 | *Streptomyces sp.* | 813 | OF | A | MK638617 |
| PN3-B10P3-7 | July 2015 | *Phyllobacterium sp.* | 1023 | OF | A | MK638372 |
| PN3-B10P3-8 | July 2015 | *Phyllobacterium sp.* | 1008 | OF | A | MK638365 |
| PN3-B10P3-9 | July 2015 | *Rhizobium sp.* | 718 | OF | A | MK638434 |
| PN3-B10P3-10 | July 2015 | *Phyllobacterium sp.* | 1025 | OF | A | MK638369 |
| PN3-B10P3-12 | July 2015 | *Kaistia sp.* | 1008 | OF | A | MK638142 |
| PN3-B10P3-13 | July 2015 | *Phyllobacterium sp.* | 1008 | OF | A | MK638360 |
| PN3-B10P3-14 | July 2015 | *Streptomyces sp.* | 1018 | OF | A | MK638615 |
| PN3-B10P3-15 | July 2015 | *Phyllobacterium sp.* | 1008 | OF | A | MK638361 |
| PN3-B10P3-16 | July 2015 | *Phyllobacterium sp.* | 1008 | OF | A | MK638362 |
| PN3-B10P3-17 | July 2015 | *Phyllobacterium sp.* | 1008 | OF | A | MK638363 |
| PN3-B10P3-18 | July 2015 | *Streptomyces sp.* | 750 | OF | A | MK638616 |
| PN3-B10P3-19 | July 2015 | *Phyllobacterium sp.* | 1008 | OF | A | MK638364 |
| PN3-B10P3-20 | July 2015 | *Phyllobacterium sp.* | 1053 | OF | A | MK638355 |
| PN3-B10P3-21 | July 2015 | *Rhizobium sp.* | 1028 | OF | A | MK638429 |
| PN3-B10P3-22 | July 2015 | *Rhizobium sp.* | 1021 | OF | A | MK638435 |
| PN3-B10P5-1 | July 2015 | *Phyllobacterium sp.* | 686 | CF | A | MK638313 |
| PN3-B10P5-2 | July 2015 | *Phyllobacterium sp.* | 1073 | CF | A | MK638339 |
| PN3-B10P5-3 | July 2015 | *Phyllobacterium sp.* | 1062 | CF | A | MK638345 |
| PN3-B10P5-4 | July 2015 | *Mycobacterium sp.* | 442 | CF | A | MK638196 |
| PN3-B10P5-5 | July 2015 | *Phyllobacterium sp.* | 1052 | CF | A | MK638337 |
| PN3-B10P5-7 | July 2015 | *Phyllobacterium sp.* | 1048 | CF | A | MK638333 |
| PN3-B10P5-8 | July 2015 | *Phyllobacterium sp.* | 1037 | CF | A | MK638368 |
| PN3-B10P5-9 | July 2015 | *Phyllobacterium sp.* | 1067 | CF | A | MK638325 |
| PN3-B10P5-10 | July 2015 | *Phyllobacterium sp.* | 1071 | CF | A | MK638326 |
| PN3-B10P5-11 | July 2015 | *Phyllobacterium sp.* | 1055 | CF | A | MK638342 |
| PN3-B10P5-12 | July 2015 | *Phyllobacterium sp.* | 1071 | CF | A | MK638323 |
| PN3-B10P5-13 | July 2015 | *Variovorax sp.* | 1050 | CF | A | MK638658 |
| PN3-B10P5-14 | July 2015 | *Phyllobacterium sp.* | 1061 | CF | A | MK638348 |
| PN3-B10P5-15 | July 2015 | *Phyllobacterium sp.* | 1027 | CF | A | MK638375 |
| PN3-B10P5-16 | July 2015 | *Ensifer sp.* | 1073 | CF | A | MK638125 |
| PN3-B10P5-17 | July 2015 | *Phyllobacterium sp.* | 1070 | CF | A | MK638332 |
| PN3-B10P5-18 | July 2015 | *Streptomyces sp.* | 1018 | CF | A | MK638645 |
| PN3-B10P5-19 | July 2015 | *Streptomyces sp.* | 970 | CF | A | MK638486 |
| PN3-B10P5-20 | July 2015 | *Phyllobacterium sp.* | 1029 | CF | A | MK638308 |
| PN3-B10P5-21 | July 2015 | *Streptomyces sp.* | 955 | CF | A | MK638485 |
| PN3-B10P5-22 | July 2015 | *Phyllobacterium sp.* | 1053 | CF | A | MK638304 |
| PN3-B10P5-23 | July 2015 | *Phyllobacterium sp.* | 708 | CF | A | MK638312 |

Supplementary Table 2: Properties of the studied soils in the treatments of the GCEF. Values represent means and standard deviations (in parenthesis) for soil moisture, pH, total carbon content (TOC), total nitrogen content (TN), mineral nitrogen (N_min_) and available phosphorus (P_DL_) of five replicates for each treatment. Treatments include conventional (CF) and organic farming (OF) under ambient (A) and future (F) climatic conditions implemented in the GCEF. Samples were taken in May and July 2015.

|  | May 2015 (BBCH 37-39) | | | | July 2015 (BBCH 75-77) | | | |
| --- | --- | --- | --- | --- | --- | --- | --- | --- |
| Treatment | **CF-A** | **CF-F** | **OF-A** | **OF-F** | **CF-A** | **CF-F** | **OF-A** | **OF-F** |
| Moisture  [% w/w] | 10.9a  (0.6) | 10.1a  (0.8) | 10.6a  (0.6) | 10.6a  (0.4) | 15.9a  (0.2) | 15.9a  (0.5) | 16.0a  (0.2) | 15.8a  (0.5) |
| pH | 6.8a  (0.4) | 6.8a  (0.4) | 6.5a  (0.6) | 6.6a  (0.5) | 6.8a  (0.4) | 6.8a  (0.5) | 6.6a  (0.6) | 6.6a  (0.5) |
| TOC  [% w/w] | 2.0a  (0.1) | 2.0a  (0.1) | 2.0a  (0.1) | 1.9a  (0.1) | 1.9a  (0.2) | 1.9a  (0.2) | 1.9a  (0.1) | 1.9a  (0.2) |
| TN  [% w/w] | 0.17a  (0.01) | 0.17a  (0.01) | 0.16a  (0.01) | 0.16a  (0.01) | 0.16a  (0.02) | 0.15a  (0.02) | 0.15a  (0.01) | 0.15a  (0.02) |
| N_min_  [mg/kg] | 13.4ab  (5.1) | 9.5b  (2.6) | 5.0c  (1.4) | 4.8c  (1.0) | 12.3ab  (2.1) | 13.7a  (1.6) | 9.6ab  (4.4) | 7.7bc  (1.2) |
| P_DL_  [mg/100g] | 7.6a  (4.1) | 8.4a  (3.7) | 6.8a  (3.7) | 6.0a  (2.5) | 7.3a  (3.2) | 8.9a  (4.3) | 7.2a  (4.2) | 6.0a  (2.2) |

Supplementary Table 3: Winter wheat yields in the GCEF in 2015. Corn and straw yields are presented for conventional and organic farming systems under ambient and future climatic conditions. Significant differences in yield between the four treatments are marked by different letters according to analysis of variance and Tukey post hoc test.

| Land use | Climate | Crop | Corn yield  in dt/ha | Straw yield  in dt/ha | Ratio corn/straw |
| --- | --- | --- | --- | --- | --- |
| Conventional Farming | Ambient | Winter wheat | 83.4 **a**  (3.2) | 69.0  (4.3) | 0.82  (0.04) |
| Conventional Farming | Future | Winter wheat | 69.0 **b**  (10.4) | 58.3  (9.7) | 0.85  (0.02) |
| Organic Farming | Ambient | Winter wheat | 82.9 **a**  (4.9) | 71.0  (4.5) | 0.86  (0.06) |
| Organic Farming | Future | Winter wheat | 74.4 **b**  (5.0) | 69.2  (6.1) | 0.93  (0.03) |

Supplementary Table 4: Number of isolated colonies cultivated on Pikovskaya medium. Plot code refers to the coding of the GCEF plots, and land use and climate treatment of each plot is indicated. The land use treatment includes conventional and organic farming, and the climate ambient and future climatic conditions. Samples were taken in May and July 2015.

| Plot | Land use | Climate | May 2015  (BBCH 37-39) | July 2015  (BBCH 75-77) |
| --- | --- | --- | --- | --- |
| B01P2 | Conventional Farming | Ambient | 20 | 19 |
| B01P4 | Organic Farming | Ambient | 22 | 22 |
| B02P3 | Organic Farming | Future | 21 | 21 |
| B02P4 | Conventional Farming | Future | 20 | 14 |
| B03P1 | Conventional Farming | Ambient | 23 | 19 |
| B03P2 | Organic Farming | Ambient | 23 | 21 |
| B04P1 | Conventional Farming | Future | 20 | 18 |
| B04P2 | Organic Farming | Future | 22 | 22 |
| B05P1 | Organic Farming | Ambient | 19 | 19 |
| B05P3 | Conventional Farming | Ambient | 21 | 22 |
| B06P2 | Conventional Farming | Future | 18 | 23 |
| B06P4 | Organic Farming | Future | 20 | 23 |
| B07P1 | Organic Farming | Future | 20 | 20 |
| B07P5 | Conventional Farming | Future | 22 | 21 |
| B08P4 | Conventional Farming | Ambient | 20 | 18 |
| B08P5 | Organic Farming | Ambient | 21 | 23 |
| B09P3 | Conventional Farming | Future | 20 | 19 |
| B09P5 | Organic Farming | Future | 20 | 21 |
| B10P3 | Organic Farming | Ambient | 18 | 20 |
| B10P5 | Conventional Farming | Ambient | 20 | 22 |

Supplementary Table 5: Bacteria cultivated on Pikovskaya medium from rhizosphere soil of winter wheat. Bacteria were identified by partial *16S rRNA* sequencing. Distribution of genera among conventional (CF) and organic farming system (OF), ambient (A) and future climate conditions (F), as well as sampling time, May and July 2015.

| Genus | Total | May 2015  (BBCH 37-39) | July 2015  (BBCH 75-77) | CF | OF | A | F |
| --- | --- | --- | --- | --- | --- | --- | --- |
| *Achromobacter* | 1 | 1 | 0 | 1 | 0 | 1 | 0 |
| *Afipia* | 1 | 0 | 1 | 1 | 0 | 0 | 1 |
| *Agrobacterium* | 30 | 10 | 20 | 21 | 9 | 24 | 6 |
| *Agromyces* | 2 | 1 | 1 | 2 | 0 | 2 | 0 |
| *Arthrobacter* | 8 | 8 | 0 | 3 | 5 | 3 | 5 |
| *Bacillus* | 21 | 12 | 9 | 5 | 16 | 5 | 16 |
| *Bosea* | 1 | 0 | 1 | 1 | 0 | 0 | 1 |
| *Bradyrhizobium* | 3 | 2 | 1 | 1 | 2 | 1 | 2 |
| *Burkholderia* | 3 | 2 | 1 | 0 | 3 | 2 | 1 |
| *Buttiauxella* | 8 | 8 | 0 | 8 | 0 | 0 | 8 |
| *Caulobacter* | 2 | 1 | 1 | 2 | 0 | 1 | 1 |
| *Chitinophaga* | 2 | 2 | 0 | 2 | 0 | 0 | 2 |
| *Chryseobacterium* | 2 | 1 | 1 | 2 | 0 | 0 | 2 |
| *Clavibacter* | 1 | 1 | 0 | 1 | 0 | 1 | 0 |
| *Devosia* | 1 | 0 | 1 | 1 | 0 | 0 | 1 |
| *Dyadobacter* | 3 | 3 | 0 | 2 | 1 | 0 | 3 |
| *Dyella* | 5 | 2 | 3 | 3 | 2 | 5 | 0 |
| *Ensifer* | 11 | 1 | 10 | 6 | 5 | 6 | 5 |
| *Flavobacterium* | 4 | 4 | 0 | 2 | 2 | 2 | 2 |
| *Herbiconiux* | 1 | 0 | 1 | 1 | 0 | 0 | 1 |
| *Inquilinus* | 8 | 1 | 7 | 5 | 3 | 7 | 1 |
| *Kaistia* | 1 | 0 | 1 | 0 | 1 | 1 | 0 |
| *Leifsonia* | 7 | 1 | 6 | 6 | 1 | 7 | 0 |
| *Luteibacter* | 3 | 0 | 3 | 0 | 3 | 1 | 2 |
| *Massilia* | 1 | 1 | 0 | 0 | 1 | 1 | 0 |
| *Mesorhizobium* | 32 | 16 | 16 | 16 | 16 | 20 | 12 |
| *Microbacterium* | 7 | 2 | 5 | 4 | 3 | 4 | 3 |
| *Mucilaginibacter* | 1 | 1 | 0 | 1 | 0 | 0 | 1 |
| *Mycobacterium* | 3 | 0 | 3 | 1 | 2 | 1 | 2 |
| *Ochrobactrum* | 3 | 3 | 0 | 0 | 3 | 0 | 3 |
| *Paenarthrobacter* | 1 | 0 | 1 | 0 | 1 | 0 | 1 |
| *Pantoea* | 1 | 1 | 0 | 1 | 0 | 0 | 1 |
| *Paraburkholderia* | 2 | 1 | 1 | 0 | 2 | 1 | 1 |
| *Pedobacter* | 1 | 1 | 0 | 1 | 0 | 1 | 0 |
| *Phyllobacterium* | 184 | 37 | 147 | 96 | 88 | 77 | 107 |
| *Plantibacter* | 5 | 5 | 0 | 0 | 5 | 1 | 4 |
| *Pseudaminobacter* | 1 | 0 | 1 | 1 | 0 | 0 | 1 |
| *Pseudomonas* | 167 | 150 | 17 | 82 | 85 | 91 | 76 |
| *Rhizobium* | 44 | 20 | 24 | 19 | 25 | 29 | 15 |
| *Rhodococcus* | 5 | 5 | 0 | 2 | 3 | 3 | 2 |
| *Rugamonas* | 5 | 5 | 0 | 4 | 1 | 2 | 3 |
| *Serratia* | 2 | 2 | 0 | 1 | 1 | 1 | 1 |
| *Sphingomonas* | 1 | 1 | 0 | 1 | 0 | 1 | 0 |
| *Stenotrophomonas* | 8 | 6 | 2 | 1 | 7 | 5 | 3 |
| *Streptomyces* | 197 | 81 | 116 | 85 | 112 | 95 | 102 |
| *Tardiphaga* | 2 | 1 | 1 | 1 | 1 | 0 | 2 |
| *Tsukamurella* | 2 | 2 | 0 | 2 | 0 | 0 | 2 |
| *Variovorax* | 13 | 8 | 5 | 4 | 9 | 10 | 3 |
| Total | **817** | **410** | **407** | **399** | **418** | **412** | **405** |

Supplementary Table 6: Abundances and activity levels of dominant genera and their respective phylogenetic clusters. Numbers of isolates of each cluster are listed for May (BBCH 37-39) and July 2015 (BBCH 75-77) samples, in conventional (CF) and organic farming systems (OF), as well as under ambient (A) and future (F) climatic conditions. Mean activities ± standard deviations for phosphate solubilization, PSI 1 and PSI 2, and mean drought resistance are given for each cluster. Significant differences in phosphate solubilizing and drought resistance, between clusters are marked by different letters according to analysis of variance and Tukey post hoc test.

| **Cluster** | **May** | **July** | **CF** | **OF** | **A** | **F** | **PSI 1 in µg PO_4_^3-^** | | **PSI 2 in µg PO_4_^3-^** | | **Resistance in %** | |
| --- | --- | --- | --- | --- | --- | --- | --- | --- | --- | --- | --- | --- |
| ***Phyllobacterium*** | | | | | | | | | | | | |
|  | **33** | **142** | **94** | **81** | **73** | **102** | **456.1** | **± 16.7** | **126.6** | **± 9.7** | **61.7** | **± 1.8** |
| **C 1** | 24 | 97 | 76 | 45 | 50 | 71 | 452.3 | ± 20.2^c^ | 117.8 | ± 10.8^b^ | 58.8 | ± 1.7^ac^ |
| **C 2** | 9 | 45 | 18 | 36 | 23 | 31 | 463.3 | ± 27.9^c^ | 143.5 | ± 18.4^b^ | 67.1 | ± 3.7^a^ |
| ***Pseudomonas*** | | | | | | | | | | | | |
|  | **150** | **19** | **83** | **86** | **91** | **78** | **1027.3** | **± 51.2** | **258.8** | **± 26.8** | **37.3** | **± 2.6** |
| **C 1** | 38 | 1 | 26 | 13 | 24 | 15 | 1378.7 | ± 104.5^a^ | 433.8 | ± 69.5^a^ | 31.0 | ± 2.5^b^ |
| **C 2** | 23 | 2 | 12 | 13 | 10 | 15 | 637.81 | ± 95.6^bc^ | 142.3 | ± 32.7^bc^ | 25.0 | ± 0^bc^ |
| **C 3** | 17 | 3 | 7 | 13 | 8 | 12 | 1076.2 | ± 149.7^ab^ | 236.7 | ± 41.5^ab^ | 35.6 | ± 5^abc^ |
| **C 4** | 18 | 1 | 6 | 13 | 18 | 1 | 758.89 | ± 72^b^ | 68.1 | ± 37.8^bc^ | 39.5 | ± 9.9^bc^ |
| **C 5** | 7 | 1 | 1 | 7 | 2 | 6 | 1151.5 | ± 178.8 | 251.0 | ± 91.1 | 40.1 | ± 2.7 |
| **C 6** | 6 | 0 | 3 | 3 | 3 | 3 | 100.87 | ± 56.4 | 0.7 | ± 0.5 | NA |  |
| **C 7** | 5 | 0 | 3 | 2 | 3 | 2 | 951.77 | ± 284.4 | 196.7 | ± 109.6 | 56.7 | ± 19.7 |
| **C 8** | 5 | 0 | 4 | 1 | 1 | 4 | 2386.8 | ± 75.4 | 1028.4 | ± 113 | 41.4 | ± 7.1 |
| **C 9** | 3 | 1 | 2 | 2 | 2 | 2 | 538.43 | ± 110.9 | 88.2 | ± 19.2 | 0.0 |  |
| **C 10** | 3 | 1 | 4 | 0 | 3 | 1 | 688.32 | ± 218.2 | 126.5 | ± 126 | 43.9 | ± 14.2 |
| **C 11** | 0 | 3 | 0 | 3 | 0 | 3 | 290.92 | ± 105.9 | 0.0 | ± 0 | 0.0 |  |
| **C 12** | 1 | 2 | 1 | 2 | 3 | 0 | 1450.8 | ± 0 | 0.0 | ± 0 | 100.0 | ± 0 |
| **C 13** | 3 | 0 | 1 | 2 | 1 | 2 | 692.48 | ± 402.9 | 71.5 | ± 39.8 | 30.0 | ± 0 |
| **C 14** | 2 | 0 | 2 | 0 | 1 | 1 | 1276.7 | ± 174.1 | 105.4 | ± 10 | 46.5 | ± 20.2 |
| **C 15** | 2 | 0 | 0 | 2 | 2 | 0 | 431.25 | ± 30.4 | 1.5 | ± 0.6 | 75.4 | ± 6.7 |
| **C 16** | 1 | 1 | 2 | 0 | 1 | 1 | 1682.5 | ± 0 | 504.6 | ± 0 | 0.0 |  |
| **C 17** | 2 | 0 | 0 | 2 | 1 | 1 | 508.39 | ± 87.8 | 11.9 | ± 11.9 | 28.6 | ± 0 |
| **C 18** | 2 | 0 | 0 | 2 | 2 | 0 | 1451.7 | ± 163.6 | 483.1 | ± 21.5 | 25.4 | ± 16.3 |
| **C 19** | 1 | 1 | 1 | 1 | 1 | 1 | 584.22 | ± 0 | 85.8 | ± 51.5 | 16.7 | ± 0 |
| **C 20** | 1 | 0 | 1 | 0 | 0 | 1 | 344.33 | ± 0 | 61.0 | ± 0 | 33.3 | ± 0 |
| **C 21** | 1 | 0 | 1 | 0 | 1 | 0 | NA |  | NA |  | NA |  |
| **C 22** | 1 | 0 | 1 | 0 | 1 | 0 | NA |  | NA |  | NA |  |
| **C 23** | 1 | 0 | 0 | 1 | 0 | 1 | 1236.2 | ± 0 | 420.64 | ± 0 | NA |  |
| **C 24** | 1 | 0 | 1 | 0 | 0 | 1 | 1763.6 | ± 0 | 420.6 | ± 0 | 30.0 | ± 0 |
| **C 25** | 1 | 0 | 0 | 1 | 0 | 1 | NA |  | NA |  | NA |  |
| **C 26** | 1 | 0 | 0 | 1 | 0 | 1 | 1038.7 | ± 0 | 105.16 | ± 0 | 42.9 | ± 0 |
| **C 27** | 1 | 0 | 1 | 0 | 1 | 0 | 802.16 | ± 0 | 15.3 | ± 0 | 0.0 |  |
| **C 28** | 1 | 0 | 1 | 0 | 1 | 0 | NA |  | NA |  | NA |  |
| **C 29** | 1 | 0 | 0 | 1 | 0 | 1 | 86.082 | ± 0 | 0.2385 | ± 0 | 46.7 | ± 0 |
| **C 30** | 1 | 0 | 1 | 0 | 1 | 0 | 137.35 | ± 0 | 34.3 | ± 0 | NA |  |
| ***Streptomyces*** | | | | | | | | | | | | |
|  | **81** | **116** | **85** | **112** | **95** | **102** | **553.7** | **± 16.9** | **25.3** | **± 2.8** | **45.4** | **± 0.1** |
| **C 1** | 48 | 51 | 55 | 44 | 45 | 54 | 580.4 | ± 30.6^bc^ | 20.8 | ± 4.5^c^ | 46.3 | ± 3^b^ |
| **C 2** | 9 | 16 | 9 | 16 | 12 | 13 | 687.9 | ± 62.9^b^ | 41.8 | ± 5.5^bc^ | 45.8 | ± 6.1^bc^ |
| **C 3** | 3 | 11 | 5 | 9 | 9 | 5 | 562.8 | ± 70.9^bc^ | 24.6 | ± 6.7^bc^ | 26.4 | ± 9.3^b^ |
| **C 4** | 8 | 5 | 5 | 8 | 8 | 5 | 431.9 | ± 55^bc^ | 31.2 | ± 8.8^bc^ | 41.1 | ± 9.7^ab^ |
| **C 5** | 2 | 11 | 4 | 9 | 3 | 10 | 384.8 | ± 86.5^bc^ | 40.3 | ± 11.8^bc^ | 71.6 | ± 11.8^ac^ |
| **C 6** | 2 | 6 | 0 | 8 | 4 | 4 | 586.9 | ± 77.6 | 22.0 | ± 8.3 | 31.8 | ± 11.8 |
| **C 7** | 3 | 1 | 1 | 3 | 3 | 1 | 441.2 | ± 158 | 19.7 | ± 19.2 | 33.7 | ± 23.6 |
| **C 8** | 0 | 4 | 0 | 4 | 2 | 2 | 291.5 | ± 65.2 | 44.4 | ± 25.6 | 50.7 | ± 15.9 |
| **C 9** | 0 | 3 | 2 | 1 | 1 | 2 | 118.4 | ± 10.8 | 3.2 | ± 2.2 | 59.5 | ± 29.8 |
| **C 10** | 1 | 1 | 0 | 2 | 1 | 1 | 858.4 | ± 0 | 27.7 | ± 19.1 | 46.5 | ± 9 |
| **C 11** | 1 | 0 | 1 | 0 | 1 | 0 | 236.6 | ± 0 | 0.5 | ± 0 | 66.7 | ± 0 |
| **C 12** | 1 | 0 | 0 | 1 | 1 | 0 | NA |  | NA |  | NA |  |
| **C 13** | 1 | 0 | 0 | 1 | 1 | 0 | 504.6 | ± 0 | 0.0 | ± 0 | 50.0 | ± 0 |
| **C 14** | 1 | 0 | 1 | 0 | 1 | 0 | 34.3 | ± 0 | 0.0 | ± 0 | 0.0 |  |
| **C 15** | 0 | 1 | 0 | 1 | 0 | 1 | 292.1 | ± 0 | 2.1 | ± 0 | 81.8 | ± 0 |
| **C 16** | 1 | 0 | 0 | 1 | 1 | 0 | 596.1 | ± 0 | 3.8 | ± 0 | 61.1 | ± 0 |
| **C 17** | 0 | 1 | 0 | 1 | 1 | 0 | 774.7 | ± 0 | 0.2 | ± 0 | 65.6 | ± 0 |
| **C 18** | 0 | 1 | 0 | 1 | 1 | 0 | 309.04 | ± 0 | 8.6 | ± 0 | 100.0 | ± 0 |
| **C 19** | 0 | 1 | 1 | 0 | 0 | 1 | 504.6 | ± 0 | 8.6 | ± 0 | 0.0 |  |
| **C 20** | 0 | 1 | 0 | 1 | 0 | 1 | 68.9 | ± 0 | 6.0 | ± 0 | 66.7 | ± 0 |
| **C 21** | 0 | 1 | 1 | 0 | 0 | 1 | 596.1 | ± 0 | 46.7 | ± 0 | 44.1 | ± 0 |
| **C 22** | 0 | 1 | 0 | 1 | 0 | 1 | 381.5 | ± 0 | 95.4 | ± 0 | 30.3 | ± 0 |
